# The effects of dipeptidyl peptidase-4 inhibitors on bone fracture among patients with type 2 diabetes mellitus: A network meta-analysis of randomized controlled trials

**DOI:** 10.1371/journal.pone.0187537

**Published:** 2017-12-05

**Authors:** Jun Yang, Chao Huang, Shanshan Wu, Yang Xu, Ting Cai, Sanbao Chai, Zhirong Yang, Feng Sun, Siyan Zhan

**Affiliations:** 1 Department of Epidemiology and Biostatistics, School of Public Health, Peking University Health Science Centre, Beijing, China; 2 National Clinical Research Center of Digestive Diseases, Beijing Friendship Hospital, Capital Medical University, Beijing, China; 3 Department of Endocrinology and Metabolism, Peking University International Hospital, Beijing, China; 4 The Primary Care Unit, School of Clinical Medicine, University of Cambridge, Cambridge, United Kingdom; Universita degli Studi di Perugia, ITALY

## Abstract

**Aim:**

The association between dipeptidyl peptidase-4 inhibitors (DPP-4is), a class of anti-diabetes, and bone fracture in patients with type 2 diabetes mellitus (T2DM) is unknown. This meta-analysis aimed to systematically evaluate the effects of DPP-4is on bone fracture in T2DM patients.

**Methods:**

We searched the Cochrane Library, Embase, Medline and ClinicalTrials.gov from inception through April 28th, 2016 to identify randomized controlled trials (RCTs) that compared DPP-4is with placebo or other anti-diabetes in T2DM patients. RCTs lasting more than 12 weeks and having data on bone fracture were included. We conducted random-effects meta-analysis to estimate odds ratios (ORs) and their 95% confidence intervals (CIs), and network meta-analysis (NMA) to supplement direct comparisons. Predictive interval plot and node-splitting method were used to evaluate the heterogeneity and inconsistency for NMA, while the funnel plot was applied to explore publication bias. Besides, study quality was assessed according to Cochrane risk of bias tool.

**Results:**

We identified 75 RCTs with a total of 70,207 patients and 11 treatments: interventions included 5 DPP-4is (alogliptin, linagliptin, saxagliptin, sitagliptin, vildagliptin), while controls included placebo and 5 other anti-diabetes (sulfonylureas, glucagon-like peptide-1 receptor agonists, metformin, thiazolidinediones, sodium-glucose co-transporter 2 inhibitors). In the NMA, the risk of fracture for alogliptin tended to decrease when versus placebo (OR, 0.51; 95% CI, 0.29 to 0.88). Besides, aloglitpin had a lower risk compared with linagliptin (OR, 0.45; 95% CI, 0.20 to 0.99) and saxagliption (OR, 0.46; 95%CI, 0.25 to 0.84); the risk was higher with saxagliptin when versus sitagliptin (OR, 1.90; 95% CI, 1.04 to 3.47) and sulfonylureas (OR, 1.98; 95% CI, 1.06 to 3.71). In the direct pairwise meta-analysis, alogliptin was associated with a non-significant tendency to reduction of bone fracture compared with placebo (OR, 0.54; 95% CI, 0.29 to 1.01). Ranking probability analysis indicated alogliptin decreased the risk of bone fracture most with a probability of 76.3%.

**Conclusion:**

Alogliptin may be associated with a lower risk of bone fracture compared with placebo, linagliptin, or saxagliptin, while other anti-diabetes did not seem to have an association with the risk of bone fracture.

## Introduction

DPP-4is is a new class of anti-diabetes which can prevent the rapid degradation of glucose-dependent insulinotropic polypeptide (GIP) and glucagon-like peptide 1(GLP-1) through inhibition of DPP-4 [1], thus enhancing insulin secretion [2]. At present, at least five DPP-4is sitagliptin, vildagliptin, saxagliptin, linagliptin and alogliptin ordered by time-to-market, are authorized on the market.

T2DM is increasingly researched and considered to be a risk factor for bone fracture due to its complications and comorbidities [3, 4]. Although detailed pathogenic mechanisms are unknown, there are some possible explanations such as direct variation in bone mineral density [5], the action of osteocalcin and adiponectin [6], fragility fractures caused by impaired bone quality [7] or just indirect impact from microvascular complications [8]. Therefore, it is critical for anti-diabetes to at least not increase the risk of bone fracture. Traditional anti-diabetes like TZDs, were reported having adverse impacts on bone health [9] and have been researched much. As for DPP-4is, studies showed it may have more effects on bone metabolism than traditional ones [10, 11] and its positive impact on the balance of vitamin D has also been revealed in recent studies [12].

To reveal the association between bone fracture and DPP-4is, more and more clinical trials [13, 14], observational studies [15] and meta-analysis [16, 17] were performed. However, the results were yet inconsistent. The clinical trial [13] indicated treatment with vildagliptin for 1 year was not associated with changes in markers of bone resorption and calcium homeostasis in patients with T2DM and mild hyperglycemia. Besides, the observational study [15] showed that treatment with saxagliptin in older T2DM patients was not associated with an increased risk of fractures and DPP-4 inhibitor use was not associated with fracture risk compared to no anti-diabetes users and to non-insulin anti-diabetic drug (NIAD) users, respectively. Whereas, in the 20-study pooling analysis [16], the incidence rates per 100 person-years for bone fracture was higher with saxagliptin versus control including placebo-controlled and active-comparator (RR, 1.81; 95% CI, 1.04 to 3.28). Mamza et al [17] indicated there was no significant association of fracture events with the use of DPP-4 inhibitor when compared with placebo or an active comparator, while Monami et al [18] held the view that DPP-4 inhibitor could reduce the risk of fracture using a meta-analysis.

In addition, all of the previous studies had some limitations. Some may conduct a less believable conclusion due to particular characteristics of study population (i.e. mild hyperglycemia) or too short duration of DPP-4is use. Furthermore, few studies included almost all kinds of DPP-4is simultaneously to assess bone fracture profiles compared with different active comparators separately. Besides, none evaluated the rank probability of different drugs on bone fracture profiles before. Finally, it is meaningful to undertake an updated meta-analysis incorporating recently published robust RCTs which concentrated on DPP-4 inhibitors.

Hence, we collected all randomized controlled trials (RCTs) in which DPP-4is were compared with placebo or traditional anti-diabetes. Using pairwise meta-analysis to summarize current evidence for DPP-4is on bone fracture in patients with T2DM and an additional NMA was used to assess the robustness of the pairwise meta-analysis by combining both direct and indirect evidence.

## Methods

The review protocol has been registered on the PROSPERO website *https://www.crd.york.ac.uk/PROSPERO/* of which the registration number was CRD42015020399. Detailed information can be found on the website. This study was conducted according to the PRISMA guideline for systematic review and meta-analysis (see S1 File).

### Search strategy

The Cochrane Library, Embase, Medline were searched from inception to April 28^th^, 2016. The following search strategy for Ovid-Medline was adapted for other databases:

1. exp (DPP-4 inhibitors or dipeptidyl peptidase 4 inhibitor)/
2. (DPP IV or DPP-4 or dipeptidyl peptidase 4).tw.
3. (januvia or juvisync or janumet or galvus or equatablets or eucreas or onglyza or kombiglyzexr or nesina or liovel or ondero or jentadueto).tw.
4. (sitagliptin phosphate or vildagliptin or saxagliptin or alogliptin or linagliptin or teneligliptin or anagliptin or gemigliptin or trelagliptin).tw.
5. randomized controlled trial.pt.
6. controlled clinical trial.pt.
7. randomized.ab.
8. placebo.ab.
9. drug therapy.fs.
10. randomly.ab.
11. trial.ab.
12. groups.ab.
13. 5 or 6 or 7 or 8 or 9 or 10 or 11 or 12
14. exp animals/ not humans.sh
15. 13 not 14
16. (Diabetes Mellitus, Type 2).tw.
17. (1 or 2 or 3 or 4) and 15 and 16

In addition, similar search strategy was also applied to some completed but unpublished trials from www.clinicaltrials.gov website.

### Study selection

Only randomized controlled trials comparing DPP-4is with placebo or other active anti-diabetes in T2DM patients with available data on bone fracture events were included in the analysis. All trials were with durations of ≥ 12 weeks. Any ongoing or completed studies without available results on the website of clinicaltrials.gov were excluded from the analysis.

The primary outcome was bone fracture irrespective of fracture sites. In the whole 211 trials, we only included studies which reported bone fracture events in at least one comparable group to the main analysis. Furthermore, for studies having “zero-event” group, the constant continuity correction method was used with addition of a correction factor of 0.5 to the number of events and non-events in both treatment groups [19]. The eligibility of studies for inclusion and exclusion criteria was assessed independently by four reviewers (SSW, JY, TC and FS) in duplicate.

### Data extraction and quality evaluation

Data were extracted using ADDIS software v1.16.5[20] with details of the trials (author, publication year, sample size, trial duration, types of intervention and control), population characteristics (background therapy, diabetes duration, age, baseline level of HbA1c), reported outcomes (number of bone fracture events in each treatment group) and information on methodology. Four investigators (SSW, JY, TC and YX) extracted data independently, in duplicate. Any discrepancies were resolved by consensus between the two independent reviewers or by a senior investigator (FS).

Quality of studies was assessed according to Cochrane risk of bias tool [21], including method for random sequence generation, allocation concealment, blinding of patients and personnel, blinding of outcome assessment, incomplete outcome data, selective reporting and company funding.

### Data analysis

#### Methods for direct treatment comparisons

Traditional pairwise meta-analysis was performed using DerSimonian-Laird random effects model [22]. Odds ratios (ORs) and their 95% confidence intervals (CIs) for bone fracture were calculated as effect measures. For studies that did not report intention-to-treat, we analyzed outcomes as all-patients randomized and used the original groups being denominator to calculate rates. The I^2^-statistic was calculated as a measure of the proportion of the overall variation that is attributable to between-study heterogeneity [23]. An I^2^ value of 50% was considered to indicate significant heterogeneity between trials [24].

#### Methods for indirect and network meta-analysis

A random-effects NMA based on a frequentist framework [25] was used to evaluate the relative effectiveness of DPP-4is and other anti-diabetes on bone fracture, allowing both direct and indirect evidence to be taken into account simultaneously. Odds ratio and its 95% CI for bone fracture was summarized.

The surface under the cumulative ranking curve (SUCRA) [26] and mean ranks were applied to estimate ranking probabilities for all treatments from which we obtained a treatment hierarchy. SUCRA can be regarded as the percentage of efficacy of a treatment on the outcome (bone fracture) that would be ranked first without uncertainty, which is equal to 1 when the treatment is certain to be the best and 0 when it is certain to be the worst.

Besides, node-splitting method was applied to evaluate the presence of inconsistency which indicated if the information of both sources of evidence is similar enough to be combined [27]. In each comparison, difference in coefficient and standard error between direct and indirect estimations was calculated to assess the presence of inconsistency. Inconsistency was defined as disagreement between direct and indirect evidence with a p value greater than 0.05.

Predictive interval plot that incorporate the extent of heterogeneity was used to evaluate the extent of uncertainty in the estimated effect size for the NMA [28]. Uncertainty affected by heterogeneity was defined as disagreement between the confidence intervals of relative treatment effects and their predictive intervals.

#### Sensitivity analysis

A predefined sensitivity analysis was carried out to determine the influence of trials which were not report the fracture event in both arms on effect size. Thus, instead of only including trials which reported bone fracture events in at least one comparable group, all the trials were used to conduct a NMA.

#### Subgroup analysis

A predefined subgroup analysis was carried out to determine the influence of double blind trials, mean age of population, trial duration, T2DM duration and background therapy on effect size.

#### Funnel plot and publication bias

The difference between the observed effect size and comparison-specific summary effect for each study was calculated. Then this variable was regressed on standard error, thus a simple linear regression line added in the funnel plot could help us explore visually if there is a publication bias [29].

All analyses were performed with Stata 13.0 using the “mvmeta”, “intervalplot”, “netfunnel”, “networkplot”, “network sidesplit” commends and so on [30] and R 3.3.0 (transforming data).

## Results

### Study characteristics and evidence network

**Fig 1:**
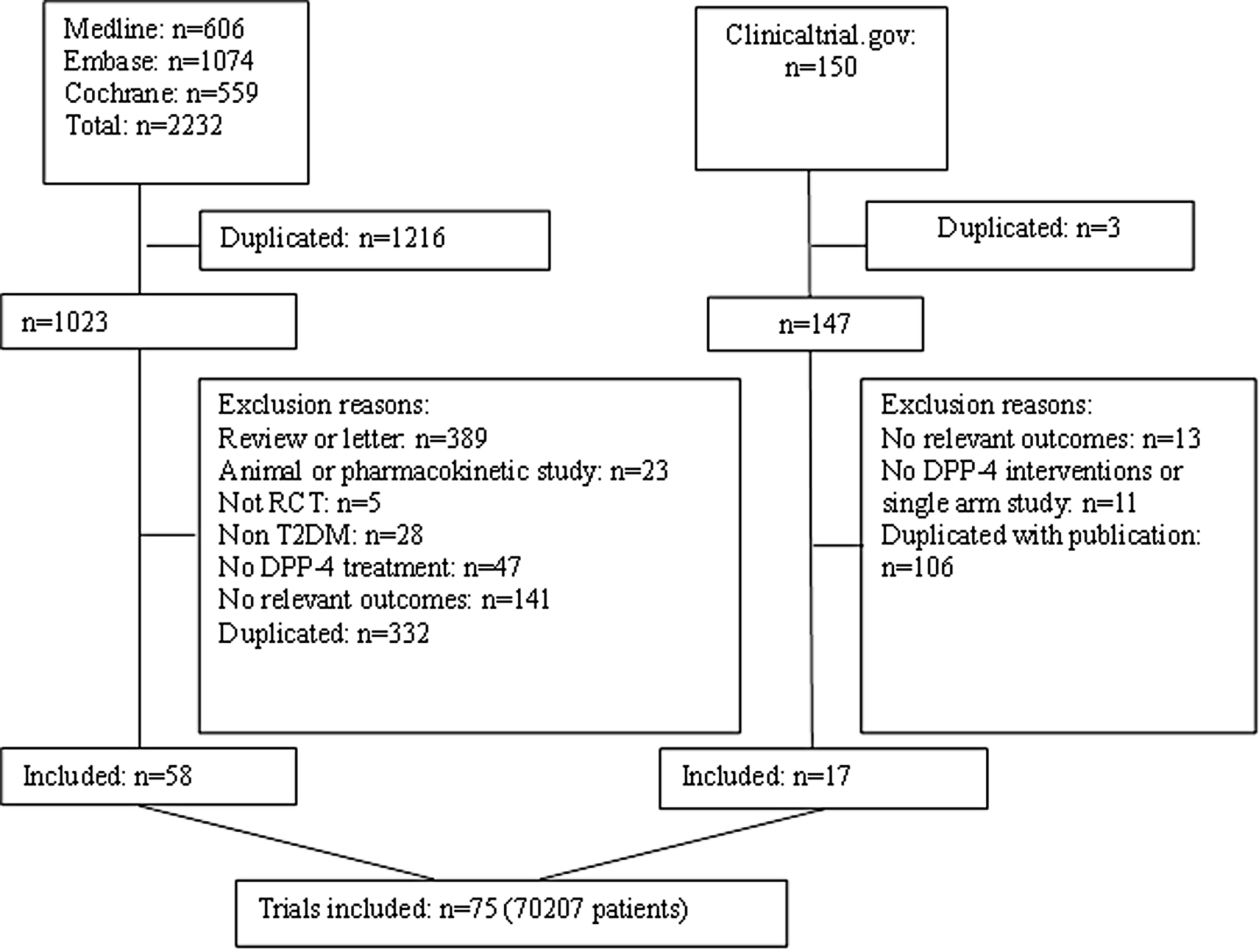
Flow chart of studies considered for inclusion, RCT = randomized controlled trial.

75 trials involving 11 treatments met the inclusion criteria, enrolling 70207 patients. Flow chart of trials selection was shown in Fig 1.

### Study characteristics

Table 1 summarized the characteristics of included 75 trials. Publication year varied from 2006 to 2016. Trial duration ranged from 12 to 206 weeks with a median follow-up of 26 weeks. The average age of participants was 57.8 years [standard deviation (SD): 5.5], varied from 49.7 to 74.9 years. The median diabetes duration at baseline was 7.1 years. And the median baseline HbA1c level was 8.1% (SD: 0.5%). Besides, patients of 86.8% trails had background treatment, more detailed information can be found in Table 1. We focused on the most five common DPP-4 inhibitors (alogliptin, linagliptin, saxagliptin, sitagliptin and vildagliptin) which were studied in 8, 14, 13, 34 and 6 trials respectively. Besides, 47 trials were placebo-controlled and 21 compared with active controls, while 7 trials included both placebo and active comparator arms. Active comparators included 5 traditional anti-diabetes (SU, GLP-1RAs, Met, TZDs and SGLT-2) which were served as control groups together with placebo.

**Table 1:** Characteristics of the 75 studies included in the NMA.

Study ID	Investigational treatments	Size	Background therapy	Trial duration (w)	Baseline information		
					Age (yrs)	HbA1c (%)	Years of T2DM
Arjona Ferreira JC 2013	Sitagliptin, SU	423	NO	54	64.2	7.8	NR
Arjona Ferreira JC 2013	Sitagliptin, SU	129	NO	54	59.5	7.8	17.5
Aschner P 2010	Sitagliptin, Met	1050	NO	24	56.0	7.3	2.4
Barnett AH 2013	Saxagliptin, Placebo	455	Insulin/Insulin+ Met	52	57.2	8.7	12.0
Barnett AH 2012	Saxagliptin, SU	455	Insulin/Insulin+ Met	24	57.2	8.7	12.0
Barnett AH 2013	Linagliptin, Placebo	241	OAD	24	74.9	7.8	NR
Barzilai N 2011	Sitagliptin, Placebo	206	OAD	24	71.9	7.8	7.1
CANTATA-D, Lavalle-Gonzalez FJ 2013	Sitagliptin,SLGT-2,Placebo	1284	Met	26	55.4	7.9	6.9
CANTATA-D2, Schernthaner G 2013	Sitagliptin,SLGT-2	755	Met+SU	52	56.7	8.1	9.6
Charbonnel B 2006	Sitagliptin, SU	701	Met+TZD	104	54.5	8.0	6.2
DeFronzo RA 2012	Alogliptin, TZD, Placebo	1554	Met	26	54.5	8.6	6.2
Dobs AS 2013	Sitagliptin, Placebo	262	Met+TZD	54	54.5	8.8	9.3
ENDURE, NCT00856284 2013	Alogliptin, SU	2639	Met	104	55.4	7.6	5.5
Fonseca V 2013	Sitagliptin, Placebo	313	Met+TZD	26	56.1	8.7	9.8
Frederich R 2012	Saxagliptin, Placebo	294	NO	24	55.0	7.9	1.7
Göke B 2013	Saxagliptin, SU	858	Met	104	57.6	7.7	5.5
Haak T 2013	Linagliptin, Placebo	395	Met	54	55.8	7.5	NR
Jadzinsky M 2009	Saxagliptin, Placebo	1306	Met	24	52.0	9.5	1.7
Kashiwagi A 2011	Sitagliptin, Placebo	134	TZD	12	58.4	8.1	7.9
NCT00121667 2014	Saxagliptin, Placebo	743	Met+TZD	206	54.6	8.1	NR
Raz I 2008	Sitagliptin, Placebo	190	Met	18	54.8	9.2	NR
NCT00601250 2009	Linagliptin, Placebo	700	Met	24	56.5	8.1	NR
NCT00602472 2014	Linagliptin, Placebo	1055	Met+SU	24	58.1	8.1	NR
NCT00661362 2012	Saxagliptin, Placebo	570	Met	24	54.1	7.9	NR
Arechavaleta R 2011	Sitagliptin, SU	1035	Met	30	56.3	7.5	NR
Henry RR 2014	Sitagliptin, Placebo	1615	Met+TZD	54	NR	NR	NR
NCT00798161 2010	Linagliptin, Placebo	857	NO/Met	24	55.2	8.9	NR
Sheu WHH 2015	Linagliptin, Placebo	1261	Insulin	52	60.0	8.6	NR
GENERATION,Schernthaner G 2015	Saxagliptin, SU	720	Met	52	72.6	7.6	7.6
Yoon KH 2012	Sitagliptin, Placebo	520	TZD	24	50.9	9.5	2.1
NCT01076088 2015	Sitagliptin, Placebo	617	Met	24	52.5	8.6	NR
NCT01183013 2014	Linagliptin, Placebo	763	TZD	54	57.3	8.1	NR
NCT01204294 2012	Linagliptin, Met	574	SU/TZD/a-Glu	52	60.9	8.0	NR
NCT01215097 2013	Linagliptin, Placebo	305	Met	24	55.5	8.0	NR
Ji L,2015	Linagliptin, Placebo	689	Met	14	53.0	NR	NR
Mathieu C 2015	Sitagliptin, Placebo	658	Insulin+Met	24	58.8	NR	13.5
Nowicki M 2011	Saxagliptin, Placebo	170	OAD	52	66.5	8.3	16.7
Olansky L 2011	Sitagliptin, Placebo	1250	Met	44	49.7	9.9	3.4
Pratley RE 2009	Alogliptin, Placebo	500	SU	26	56.5	NR	7.7
Prato S 2011	Linagliptin, Placebo	503	OAD	24	55.7	8.0	NR
Raz I 2006	Sitagliptin, Placebo	741	NO	24	54.2	8.0	4.5
Roden M 2015	Sitagliptin,SLGT-2,Placebo	899	NO	76	55.0	NR	NR
Rosenstock J 2006	Sitagliptin, Placebo	353	TZD	24	56.2	8.0	6.1
Rosenstock J 2009	Saxagliptin, Placebo	401	Met	24	53.5	7.9	2.6
Rosenstock J 2013	Alogliptin, SU	441	NO	52	69.9	7.5	6.1
Ross SA 2012	Linagliptin, Placebo	491	Met	12	58.6	8.0	NR
SAVOR-TIMI 53, Raz I 2014	Saxagliptin, Placebo	16492	Insulin/ Met/SU	109	65.0	8.0	10.3
Vilsboll T 2010	Sitagliptin, Placebo	641	Insulin	24	57.8	8.7	12.5
Wainstein J 2012	Sitagliptin, TZD	517	Met/NO	32	52.3	8.9	3.2
EXAMINE, White WB 2013	Alogliptin, Placebo	5380	OAD	76	61.0	8.0	7.2
Goldstein BJ 2007	Sitagliptin, Placebo	1091	Met	104	53.5	8.8	4.5
Pratley RE 2014	Alogliptin, Placebo	334	NO	26	53.1	NR	4.0
Pratley RE 2014	Alogliptin, Placebo	450	Met	26	53.9	NR	4.1
Takihata M 2013	Sitagliptin, TZD	115	Met/SU/Met+SU	24	60.5	7.4	NR
Gallwitz B 2012	Linagliptin, SU	1551	Met	104	59.8	7.7	NR
Rosenstock J 2015	Saxagliptin,SLGT-2	266	Met	24	54.5	9.0	7.8
Rosenstock J 2015	Saxagliptin, Placebo	268	Met+SGLT-2	24	53.5	8.9	7.3
Hirose T 2015	Vildagliptin, Placebo	156	Insulin, Met	12	59.3	8.1	12.9
Mita T 2016 Hollander PL 2011 Charbonnel B 2013 Nauck M 2007 AWARD-5, Weinstock RS 2015 Roden M 2013 Takeshita Y 2015 NCT01098539 2014 Moses RG 2015 NCT01545388 2014 HARMONY3 2014	Sitagliptin, Placebo Saxagliptin, Placebo Sitagliptin, Liraglutide Sitagliptin, SU Sitagliptin, Dulaglutide Sitagliptin,SGLT-2,Placebo Vildagliptin, Liraglutide Sitagliptin, Albigutide Sitagliptin, Placebo MET, Placebo Sitagliptin, Placebo	282 565 653 1172 921 899 122 495 422 337 403	OAD TZD SU Met Met NO Sitagliptin OAD MET+SU Sitagliptin MET	104 76 26 104 104 24 12 52 24 26 156	63.7 54 57.3 56.7 54 55 64.7 63.3 54.9 59.6 54.8	8.1 8.3 8.2 7.7 8.1 NA 8 NA 8.4 7.51 NA	17.3 5.2 7.9 6.4 7 NA NA NA NA NA NA
Bosi 2011	Alogliptin, Pioglitazone	803	Met+ TZD	52	55.0	8.3	7.2
Bosi E 2009	Vildagliptin, Placebo	879	Met	24	52.8	8.7	6.1
Fonseca 2007	Vildagliptin, Placebo	296	Insulin	24	59.2	8.4	14.7
Pan C 2012	Vildagliptin, Placebo	438	Met	24	54.2	8.1	5.0
Scherbaum WA 2008	Vildagliptin, Placebo	306	NO	52	63.3	6.7	2.6
McGill JB 2014	Linagliptin, Placebo	133	SU	52	64.4	8.2	NA
Ferrannini E2013	Sitagliptin, Placebo	444	Met	78	58.6	8.1	NA

Note: NR: not report; Y: yes; N: no; OAD: oral anti-diabetic drugs; DPP-4I: including all kinds of DPP-4 (dipeptidyl peptidase-4) inhibitors; SGLT-2: Sodium-Glucose co-Transporter 2; Met: Metformin; SU: sulphanylureas; a-GLU: alpha-glucosidase inhibitor; TZD: thiazolidinediones. CANTATA-D Trial: CANagliflozin Treatment and Trial Analysis-DPP-4 Inhibitor Comparator Trial; CANTATA-D2: CANagliflozin Treatment And Trial Analysis-DPP-4 Inhibitor Second Comparator Trial; ENDURE: Efficacy and Safety of Alogliptin Plus Metformin Compared to Glipizide Plus Metformin in Patients With Type 2 Diabetes Mellitus; GENERATION: Saxagliptin Compared to Glimepiride in Elderly Type 2 Diabetes Patients, With Inadequate Glycemic Control on Metformin; EXAMINE: Examination of Cardiovascular Outcomes with Alogliptin versus Standard of Care; HARMONY3: A Study to Determine the Safety and Efficacy of Albiglutide in Patients With Type 2 Diabetes.

According to Cochrane risk of bias tool, the methods used for allocation concealment and blinding of outcome assessment were not clearly stated in some conditions (13.3% and 56.0% were unclear respectively). Conversely, the methods for randomization, blinding of participants and personnel, incomplete outcome data and selective reporting were appropriately described in almost all cases (100.0%, 100.0%, 97.3% and 97.3%, respectively), although 9.3% (7/75) of trials were open label. Additionally, 93.3% (70/75) of trials were funded by company, only 2.7% (2/75) did not report the funding sources (See S1 Table). Overall, risk of bias is respectively low.

### Evidence network

**Fig 2:**
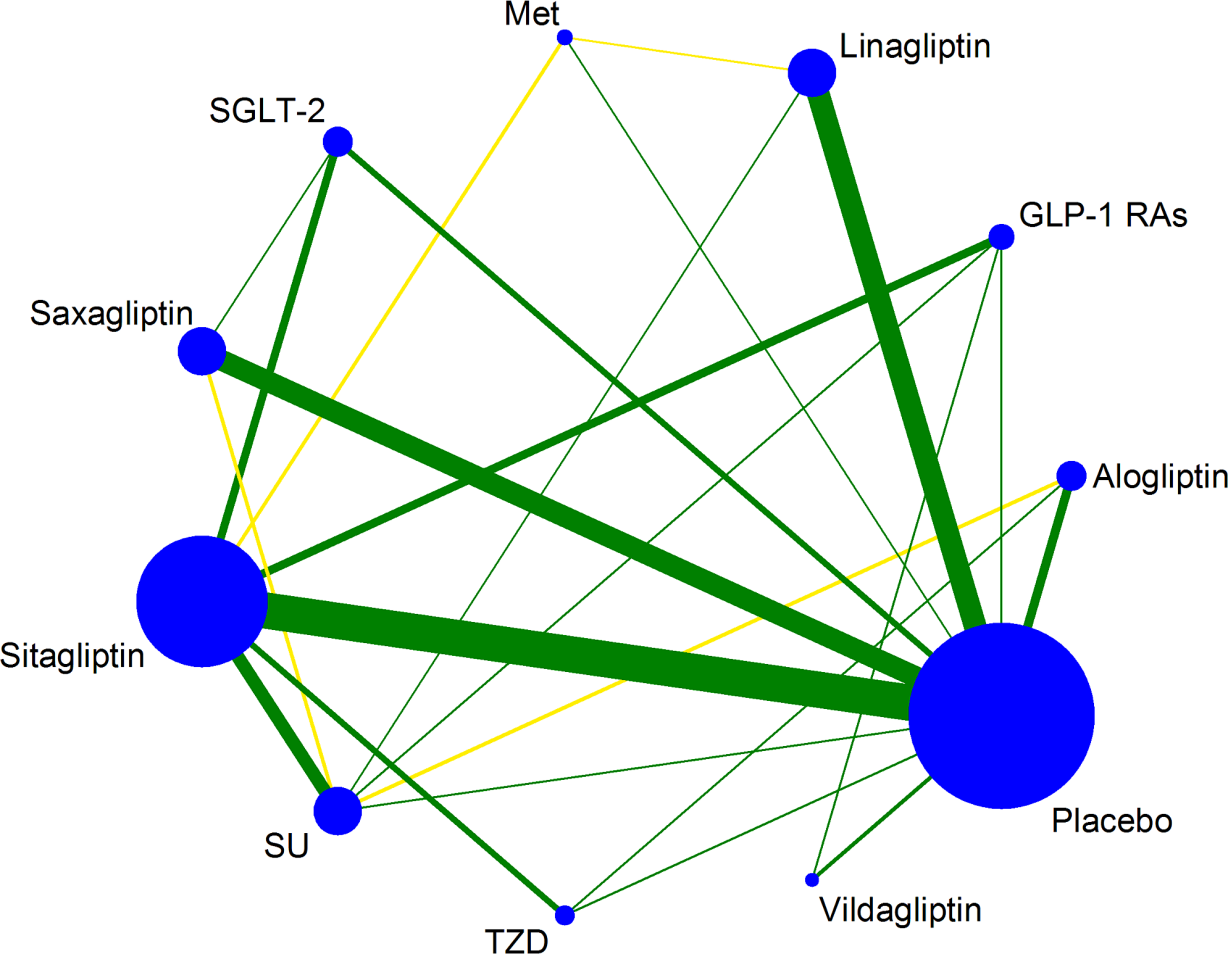
Evidence structure of eligible comparisons for NMA. Note: Lines connect the interventions that have been studied in head-to-head (direct) comparisons in the eligible RCTs. The width of the lines represents the cumulative number of RCTs for each pairwise comparison and the size of every node is proportional to the number of randomized participants (sample size). The yellow lines represent trials reporting unclear about allocation concealment, while the blue lines represent trials with low risk of allocation concealment. GLP-1RAs: Glucagon-like peptide-1 receptor agonists; SGLT-2: Sodium-Glucose co-Transporter2; Met: metformin; SU: sulphanylureas; TZD: thiazolidinediones.

90.7% (68/75) of trials were two-arm studies and the rest 7 trials were multiple-arm studies (see Table 1). Overall, 70207 patients contributed to the analysis of bone fracture (Fig 2, including 75 trials and 11 treatments).

#### Direct pairwise meta-analysis of DPP-4is on bone fracture

**Fig 3:**
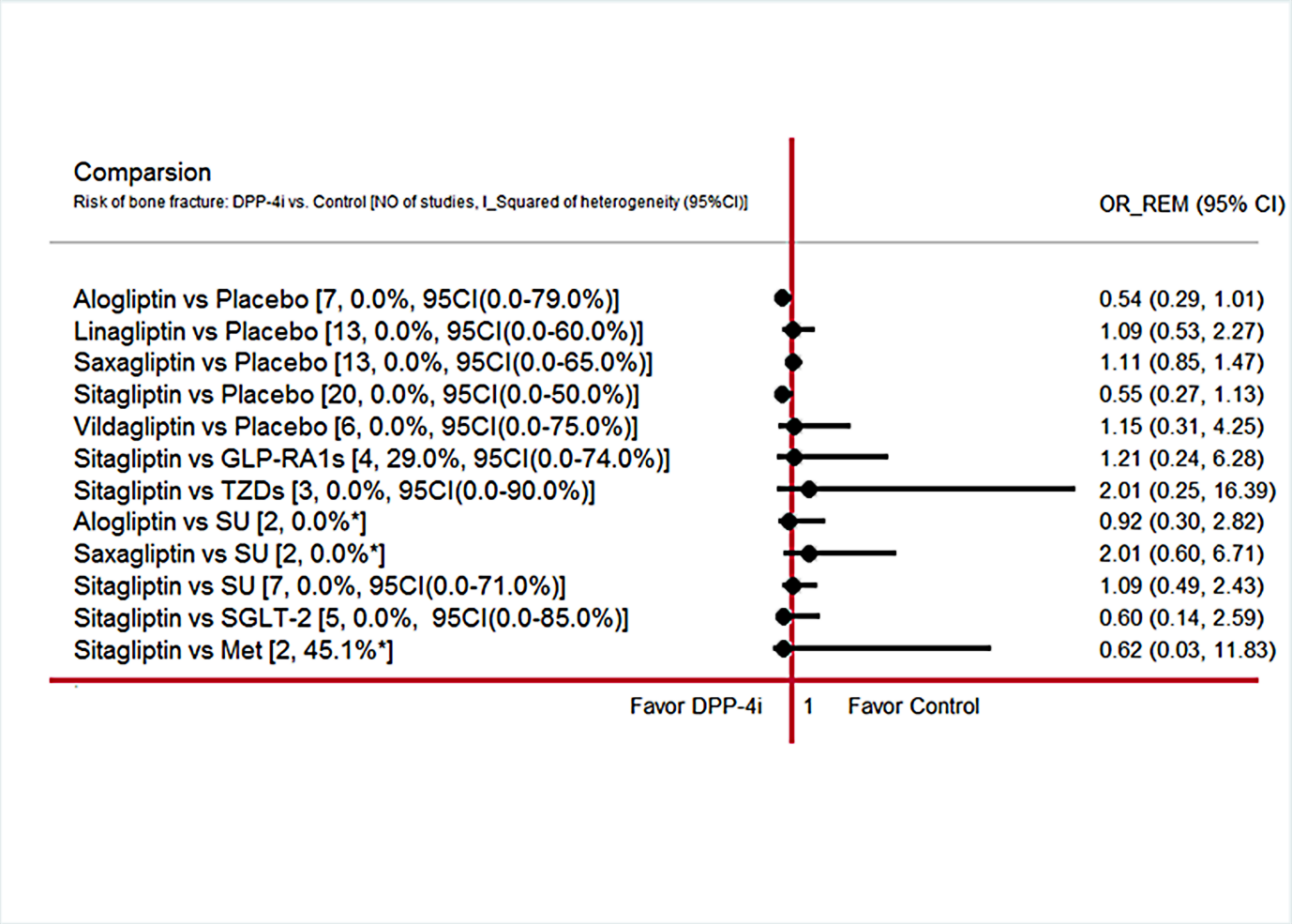
Effect of DPP-4 inhibitors regimens on bone fracture events by direct pairwise meta-analysis. Note: DPP-4i: dipeptidyl peptidase-4 inhibitor; GLP-1RAs: Glucagon-like peptide-1 receptor agonists; SGLT-2: Sodium-Glucose co-Transporter 2; Met: metformin; SU: sulphanylureas; TZD:thiazolidinediones.*notes that there was no I square’s 95% confidence interval since the degree of freedom related was less than 2(DF = NO of studies-1).

Fig 3 showed the effect of 5 kinds of DPP-4is and other anti-diabetes on bone fracture from direct pairwise meta-analysis. Alogliptin was associated with a tendency to reduction of bone fracture compared with placebo (OR, 0.54; 95% CI, 0.29 to 1.01), while it was no significant. Also, no significant effect on bone fracture following linagliptin (OR,1.09; 95%CI, 0.53 to 2.27), sitagliptin (OR, 0.55; 95%CI, 0.27 to 1.13) and other treatments was observed when compared with placebo.

#### Network meta-analysis of DPP-4is on bone fracture

**Fig 4:**
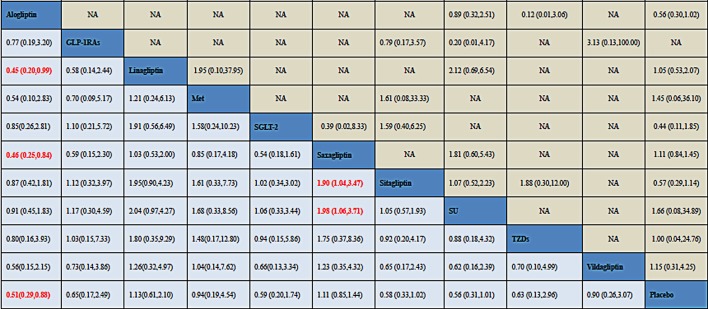
Odds ratio (OR) with 95% confidence interval (CI) of NMA for bone fracture events based on a frequentist framework. Note: Treatments were reported in alphabetical order. Results of direct comparisons were listed in the upper triangle, and the estimation was calculated as the row-defining treatment compared with the column-defining treatment. Results of NMA were listed in the lower triangle, and the estimation was calculated as the column-defining treatment compared with the row-defining treatment. NA: not available. GLP-1RAs (Glucagon-like peptide-1) receptor agonists; SGLT-2: Sodium-Glucose co-Transporter 2; Met: metformin; SU: sulphanylureas; TZDs: thiazolidinediones.

Results of direct pairwise and NMA among DPP-4is, placebo and 5 active comparators were displayed in Fig 4. For effect on bone fracture, reduction was only detected with statistical significance for alogliptin versus placebo (OR, 0.51; 95% CI, 0.29 to 0.88). No significant association with bone fracture was found for any active comparators when compared with placebo. While compared with linagliptin and saxagliptin, alogliptin also showed a decreased risk of bone fractures with ORs 0.45(0.20, 0.99) and 0.46(0.25, 0.84) respectively as well. Besides, compared with sitagliptin, saxagliptin also showed an increased risk of bone fractures with ORs equal to 1.90 (1.04, 3.47). Furthermore, an increased risk associated with saxagliptin (OR, 1.98; 95% CI, 1.06 to 3.71) was found when compared with SU.

#### Ranking probability of DPP-4is and other anti-diabetes on bone fracture

**Fig 5:**
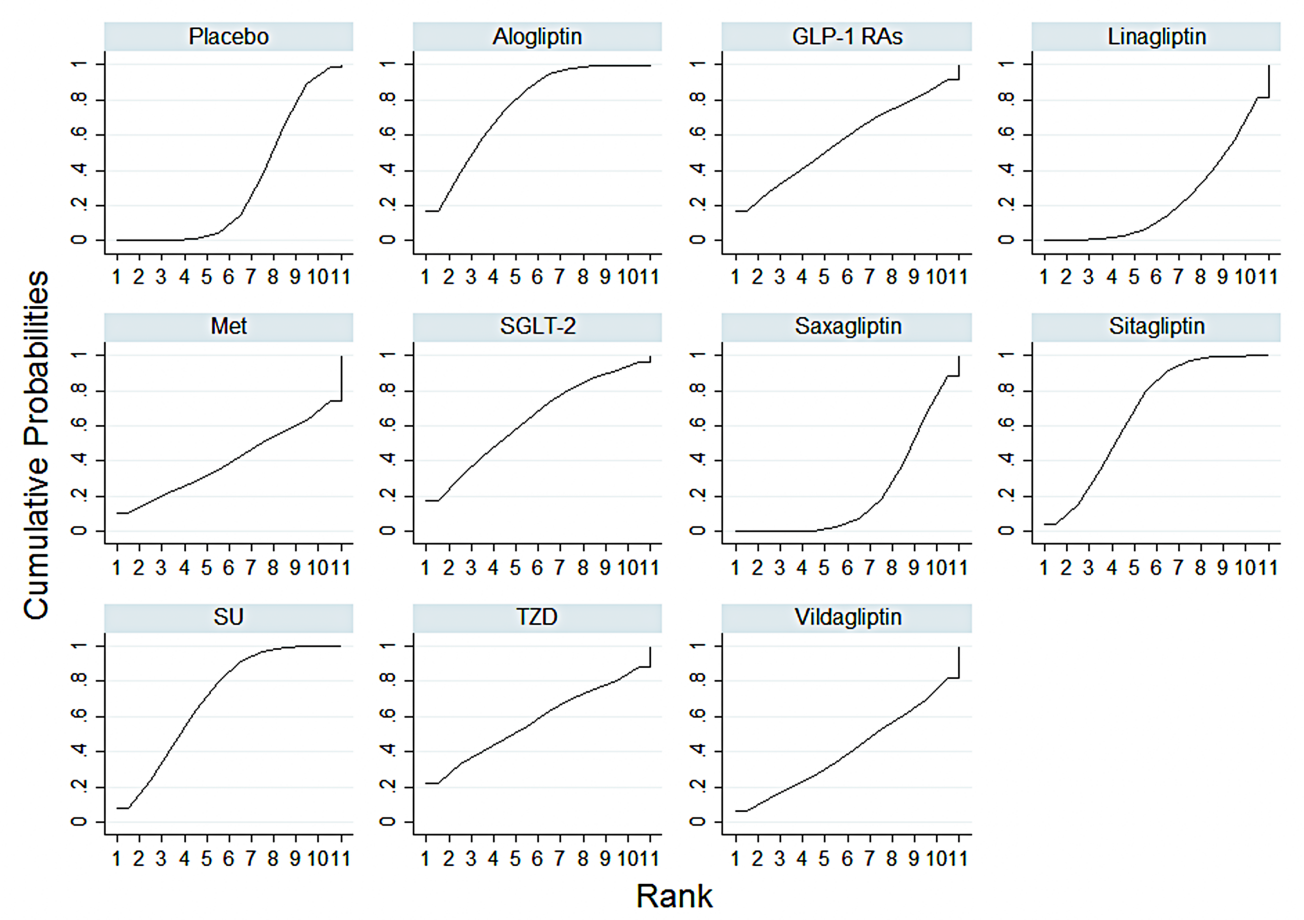
Plots of cumulative ranking probability on impact of bone fracture events (SUCRA). Note: GLP-1RAs: including all kinds of glucagon-like peptide-1(GLP-1) receptor agonists; SGLT-2: Sodium-Glucose co-Transporter 2; Met: metformin; SU: sulphanylureas; TZD: thiazolidinediones. Ranking: probability of being the best treatment, of being the second best, the third best and so on, among the 11 comparisons. SUCRA: surface under the cumulative ranking curve.

Fig 5 showed ranking probability of each treatment on bone fracture. Table 2 showed the mean values of SUCRA to provide the safety hierarchy of 11 treatments on bone fracture. Alogliptin had the lowest bone fracture risk among all eleven treatments with a probability of 76.3%, followed by SU (71.0%) and sitagliptin (67.9%).

**Table 2:** Ranking probability of different kinds of DPP-4is on bone fracture events.

Treatment	Bone fracture events	
	SUCRA	Rank
**Alogliptin**	**0.763**	**1**
**SU**	**0.710**	**2**
**Sitagliptin**	**0.679**	**3**
SGLT-2	0.633	4
TZDs	0.577	5
GLP-1RAs	0.570	6
Vildagliptin	0.403	7
Met	0.403	7
Placebo	0.313	9
Linagliptin	0.231	10
Saxagliptin	0.220	11

Note: Ranking: probability of being the best treatment, of being the second best, the third best and so on, among the 11 treatments. SUCRA: surface under the cumulative ranking curve. SU: sulphanylureas; SGLT-2: Sodium-Glucose co-Transporter 2; TZDs: thiazolidinediones; GLP-1RAs: including all kinds of glucagon-like peptide-1(GLP-1) receptor agonists; Met: Metformin.

#### Inconsistence and heterogeneity check

S2 Table showed statistical inconsistency between direct and indirect comparisons was generally low for bone fracture. All of comparisons between any two treatments were consistent, since their P values were bigger than 0.05, which meant the direct estimation of the summary effect did not differentiate from the indirect estimation.

**Fig 6:**
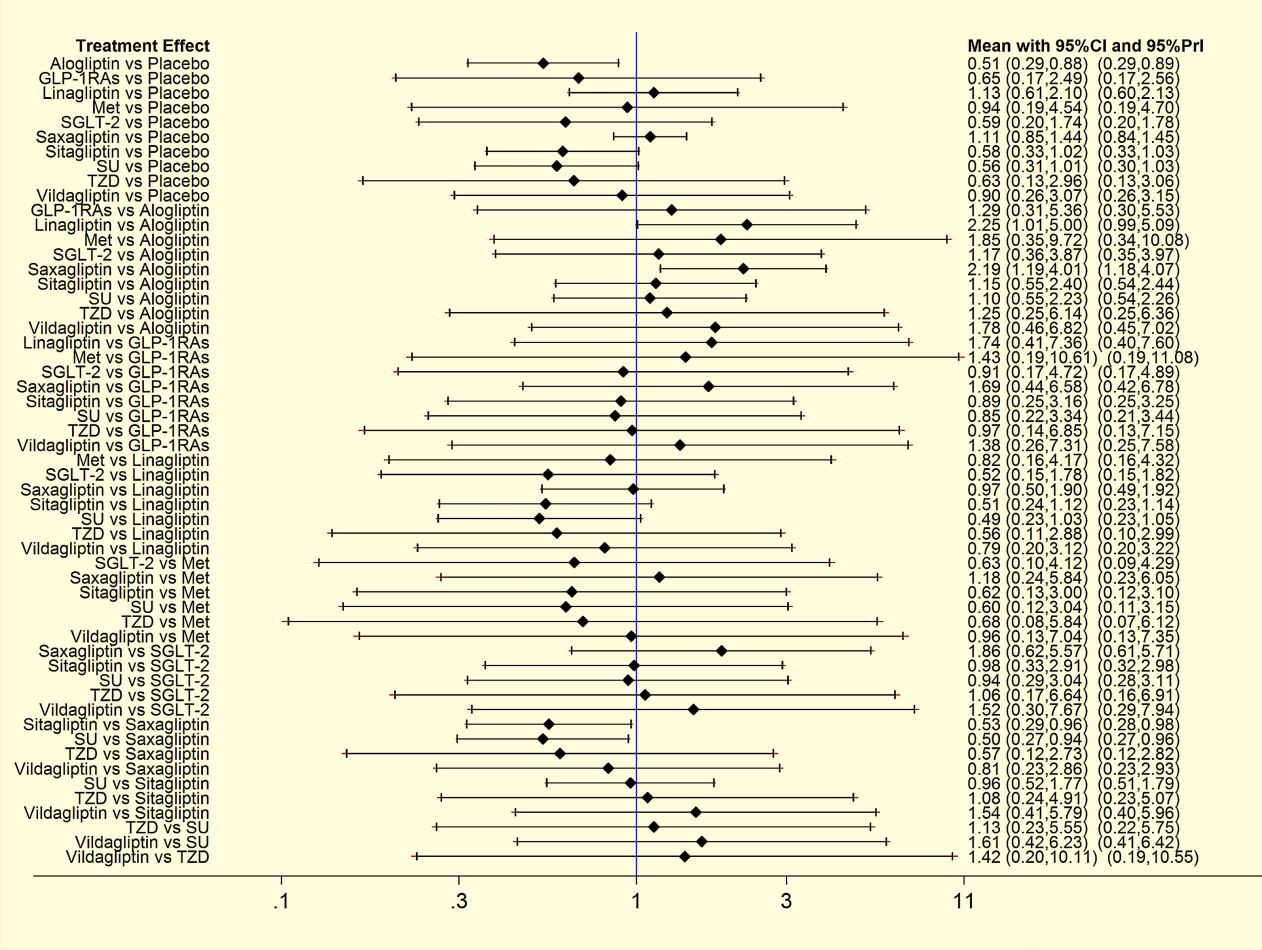
Predictive intervals plot for the DPP-4is regimens network on bone fracture events. **The graph presents the network estimates for all pairwise comparisons. Black horizontal lines represent the confidence intervals, and vertical lines represent the predictive intervals.** Note: GLP-1RAs: (Glucagon-like peptide-1) receptor agonists; SGLT-2: Sodium-Glucose co-Transporter 2; Met: metformin; SU: sulphanylureas; TZD: thiazolidinediones.

Additionally, predictive interval plot indicated that no statistical heterogeneity between the included studies was found (Fig 6). None of the comparisons was substantially affected by the estimated heterogeneity in the network, since their confidence intervals and respective predictive interval both cross the line of no effect apart from 2 comparisons. Besides, all the tau^2^ values were approximately equal to zero.

#### Sensitivity analysis

**Fig 7:**
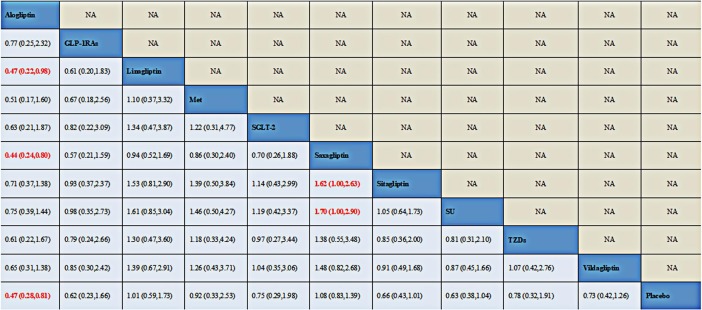
Odds ratio (OR) with 95% confidence interval (CI) of NMA for bone fracture events after enrolling both zero-events trials. Note: Treatments were reported in alphabetical order. Results of direct comparisons were listed in the upper triangle, and the estimation was calculated as the row-defining treatment compared with the column-defining treatment. Results of NMA were listed in the lower triangle, and the estimation was calculated as the column-defining treatment compared with the row-defining treatment. NA: not available. GLP-1RAs (Glucagon-like peptide-1) receptor agonists; SGLT-2: Sodium-Glucose co-Transporter 2; Met: metformin; SU: sulphanylureas; TZDs: thiazolidinediones.

Fig 7 exhibited the sensitivity analysis for NMA that when enrolled all the 211 trials, the results were consistent with previous results that alogliptin showed a beneficial effect over linagliptin, saxagliptin and placebo.

#### Subgroup analysis

Subgroup analysis for NMA was shown in S3 Table. We could see that alogliptin showed a significantly reduced risk of fracture when compared with placebo in these subgroups, including double blind group (OR, 0.51; 95% CI, 0.29 to 0.90), mean age≥60 group (OR, 0.49; 95% CI, 0.24 to 1.00), trial duration ≥52weeks group (OR, 0.55; 95% CI, 0.30 to 0.98) and no Met use group (OR, 0.48; 95% CI, 0.23 to 1.00). In addition, sitagliptin demonstrated a beneficial effect on fracture when versus placebo in mean age <60 group (OR, 0.52; 95% CI, 0.28 to 0.97) and Met using group (OR, 0.49; 95% CI, 0.24 to 0.99). The results gained from subgroup analyses were mainly consistent with previous results.

#### Funnel plot and publication bias

**Fig 8:**
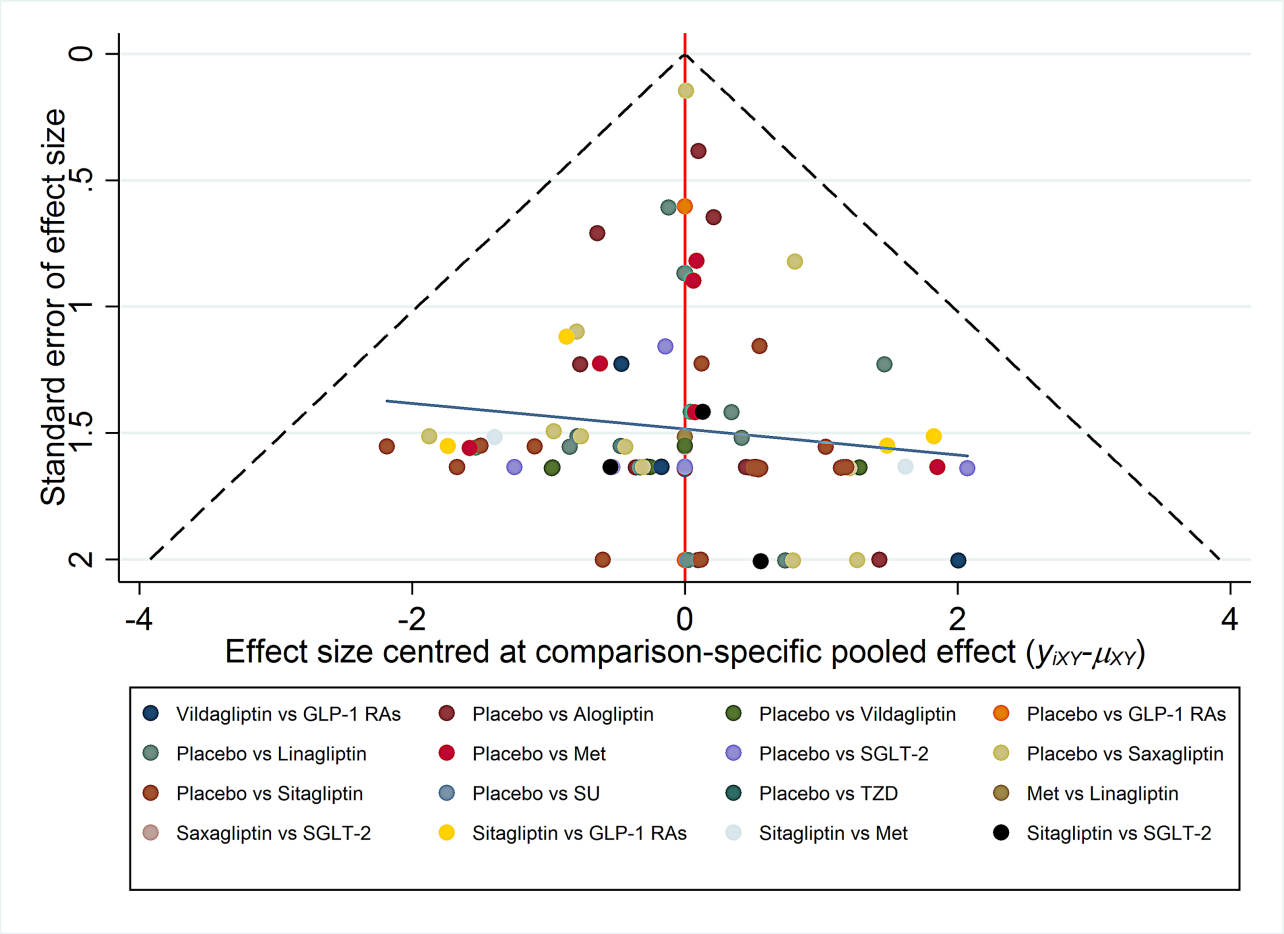
‘comparison-adjusted’ funnel plot for bone fracture events. Note: GLP-1RAs: including all kinds of glucagon-like peptide-1(GLP-1) receptor agonists; SGLT-2: Sodium-Glucose co-Transporter 2; Met: metformin; SU: sulphanylureas; TZD: thiazolidinediones.

Funnel plot for bone fracture events was shown in Fig 8. Scatters in the funnel plot were almost symmetrical visually, indicating the publication bias in the results of bone fracture events between small and large studies was relatively low.

## Discussion

Bone fracture is focused increasing attention as important endpoint among T2MD patients recently [31–33]. Our NMA suggested alogliptin seemed to associate with decreased risk of bone fracture compared with placebo, while other anti-diabetes did not show any increased risk of bone fractures. Among DPP-4is, alogliptin had a profitable effect on bone fracture when versus linagliptin and saxagliptin while sitagliptin had a beneficial effect when compared with saxagliptin. Besides, SUs was detected to reduce the risk of bone fracture in comparison with saxagliptin.

As an important member of administered DPP-4is, alogliptin showed a beneficial effect on bone fracture when compared with placebo. Other four DPP-4is did not show the superiority compared with placebo. In Yabe’s drug safety evaluation on alogliptin[34], the experiment models indicated that the incretin hormones appeared to increase bone density and showed a potentially beneficial effect for this class of agents. A recent study in Danish women revealed a critical role of GIP in increasing bone mineral density and preventing bone fractures, so there was preliminary evidence that alogliptin could increase bone density and thus reduce the risk of fractures. However, a meta-analysis of RCT trials showed all of the five DPP-4is had an indifferent impact on bone fracture [35]. The different discovery about alogliptin may result from the following reasons: first, Fu J et al just collected 62 trails including 62206 patients, while we had a larger population accounting 70207. Second, they used traditional meta-analysis only to explore the effect of every kind of DPP-4is thus could get a less robust result due to limited trails of some DPP-4is, such as anagliptin and vildagliptin, however we used the NWM method to supply indirect evidence on fracture events. Third, in the subgroup analysis, they just put all active drugs together as control group which would make the conclusion difficult to explain. Mamza et al [17] also conducted a similar work in 2015 to explore the association between DPP-4is and the incidence of fractures. They performed a traditional pairwise meta-analysis to compare DPP-4is with either active comparators or placebo and it showed that there was no significant association between DPP-4 inhibitors use and the incidence of fractures which was the same as our results in pairwise segment. Except for similar conclusion, there were several discrepancies and limitations in this research. First, they only performed pairwise meta-analysis which lacked the indirect evidence and might lead to some loss of information. Second, they compared DPP-4 inhibitors as a whole with either active comparators or placebo so that the associations between particular DPP-4 inhibitor i.e. sitagliptin and active comparator i.e. Met could not be found which were exactly released in our research. Another meta-analysis recruiting 22,055 patients showed a significant 40% reduction of fracture risk with the use of DPP-4is as compared to active treatment and placebo [18] which was the same to another review [36]. However, studies included in it did not routinely collect fractures as an outcome of interest. Besides, the conclusion acquired based on an integrated impact of 6 kinds of DPP-4is. We valued the result observed in the present study that it could be a better guideline in clinical practice when considering about a singular DPP-4 inhibitor. Preclinical researches and early clinical trials have supported the bone-protective effect of DPP-4is. An in-vivo experiment presented the clear observation supporting that sitagliptin treatment brought about increased cortical and trabecular bone volume as well as increased bone strength in diabetic animals compared with untreated diabetic animals [37]. Although, our analysis just showed only two subgroups had similar results. Besides, Scirica, BM et al [38] found alogliptin may also help to reduce bone fracture. Shah’s animal experiments in mice [39] showed that alogliptin exerted anti-atherosclerotic effect and reduced inflammation via inhibition of monocyte activation/chemotaxis which might [39] be helpful for the health of T2DM by improving the blood supply of lower limbs.

In addition, our study proposed that alogliptin and sitagliptin had a profitable effect on bone fracture when versus linagliptin and saxagliptin, respectively. However Fu J et al [35] claimed there were no statistically significant differences in the risk of fracture between every DPP-4is. The inconsistency might be possibly owing to pooling the placebo and all active comparators in a single group as control. Thus it would dilute the benefit of DPP-4is on bone fracture profiles. Other possible reasons included different sample size and dose-use of the five DPP-4is in two studies making some of the results lack of statistical power. The reason why a divergent risk of bone fractures related to different DPP-4is arose might mainly be the difference in molecular structures which could lead to the discrepancy in pharmacokinetic profiles, such as routes of metabolism [40], effective acting time, absorption and elimination pathway. For instance, having an active metabolite is a unique feature of saxagliptin compared with other DPP-4is. Whether this distinct property of saxagliptin could interact with pathways of bone metabolism and bone turnover, thus having a relatively negative impact on bone mass or strength needs to be clearly elucidated. Although all available DPP-4is in the market improve insulin secretion and glycemic control, but there may be some differences among the drugs about their effects in other tissues [41].

Meanwhile, we captured that significantly beneficial effect was found on SU when compared with saxagliptin, but not with placebo. First of all, SU act in a non-glucose-dependent fashion through the sulphonylurea receptor different from DPP-4is [42]. Previous researches pointed out the use of SU seemed to have a positive effect on bone through the enhancement of IGF-1 secretion [43]. Recent analysis of circulating bone biomarkers in a subset of patients from the ADOPT trial also demonstrated that serum CTX (s-CTX), a marker of osteoclast activity, was reduced in both male and female patients treated for 52 weeks with metformin and glyburide which meant a possibly protective effect on bone [44] Neutrally, a systematic review did not show an increased risk of falls/fractures on SU[45] yet it had several shortcomings, like insufficient events and inappropriate comparison groups. Similarly, a propensity-matched retrospective cohort study enrolling 12327 residents put forward there was no association between initiation of SU and fractures, but it was conducted on a special population, geriatric nursing home residents which could influence the final consequence [46]. Currently, only a few studies available have explored SU’s effect on bone formation, such as glimepiride, a sulfonylurea, has been shown to increase proliferation and differentiation of osteoblasts by activation of PI3K/ATK pathway causing increased ALP and osteocalcin mRNA activity in rats [47, 48].

As for GLP-1 RAs, there was not any significant results obtained in our study when compared with placebo and other anti-diabetes which was supported by the results of a cohort study [49], two meta-analysis [50, 51] and a case-control study using Danish Health Service data [52]. Nevertheless, the meta-analysis [51] including a total of 11,206 patients declared liraglutide was associated with a significant reduced risk of bone fractures, while exenatide might be associated with an increased risk via subgroup analysis. However, since we focused on DPP-4is rather than GLP-1 RAs, there were a limited number of trials relating to GLP-1 RAs enrolling in our analysis which did not allow us to explore the separate effects of every GLP-1 RAs on bone fractures. Theoretically, DPP-4is and GLP-1 RAs ought to have similar function but the former could increase both GIP and GLP-1 levels [53]. Many cellular and animal studies supported a potential role of GIP in modulating bone metabolism [54, 55], such as enhancing bone formation via stimulation of osteoblast proliferation and inhibition of apoptosis. However, the physiological role of GLP-1 on bone is less clear. Furthermore, DPP-4is not only modulate the activity of GLP-1 and GIP but also the activity of a series of other substrates, like neuropeptide Y (NPY) and substance P which also influence the activity of osteoclasts directly. Therefore, it could make sense that we obtained a disparate result on these two class drugs. Surely, further studies are needed to resolve the dispute.One more to mention, a recent meta-analysis [56] based on the population-based cohort data (n = 31466) showed that neither current DPP-4 inhibitors nor GLP-1 RAs was associated with a decreased risk of fracture which was partly opposed to our conclusion. Although its methods and results seemed to be appropriate, there were some controversial issues. First, all the studies (2 cohort studies and 2 case-control studies) included in this meta-analysis were totally published by Driessen who is exactly the author of this meta-analysis, so the representative of population and relative outcomes might be controversial. Second, the date of fracture event collection in this meta-analysis coming from the UK and Denmark was during 2007–2012 which made the data relatively outdated. Third, we did have to admit that meta-analysis based on RCTs may not have a high level of external validity due to its strictly controlled factors. However, compared to the observational study, many potential biases could be avoided in RCTs which might be better at outcome measurement with higher precision and clearer definition. In short, we preferred our results from RCTs to the real-world conclusion due to its greater number of patients and higher measurement quality. But, we also thought Driessen’s work of importance because it reflected the effect of incretins on fracture risk in the real-world situation. Hope more similar studies can be conducted to explain the truth.

## Strength and limitation

A major strength of our study is the inclusion of a substantially great number of trials and patients of DPP-4is therapies. Furthermore, we assessed the bone fracture profiles of DPP-4is compared with placebo and each active comparator separately. Besides, we found the effect of single DPP-4is reducing interference effect among them. In addition, our study provided the ranks of DPP-4is and traditional anti-diabetes on bone fracture events through NMA. Last, the age of study populations was relatively accord with the typical osteoporosis population, which might reduce an important bias in evaluation of drug effect on bone fractures.

Of course, we should acknowledged that the present results must be considered with caution because the relatively low number of trials designing to evaluate the effect of DPP-4is on fracture events and reporting data of fracture could result in the limited robustness of the current conclusion. Additionally, it was indeed difficult to visually assess the publication bias due to the small quantity of included studies, although we performed comparison-adjusted funnel plot. Another limitation is insufficiently long durations of some trails which made us cannot obtain the long-term effect of drugs on fracture. Although some studies insisted there were no statistically significant differences in the risk of fracture according to the length of follow-up (P = 0.35). Meanwhile, we did not have access to original data for any of these trials, making the statistically more powerful individual patient data meta-analysis, particularly analyses by different baseline levels of bone status, use of non-hypoglycemic agents as well as some other demographic or anthropometric characteristics, unavailable.

## Conclusion

In conclusion, our NMA provided some information on differences among DPP-4is, traditional anti-diabetes and placebo on bone fracture. Alogliptin seemed associated with decreased risk of bone fracture compared with placebo, while other drugs did not show any increased risk of bone fractures. Among DPP-4is, alogliptin had a profitable effect on bone fracture when versus linagliptin and saxagliptin, while sitagliptin also had a beneficial effect when compared with saxagliptin. Although bone fracture was not the principle concerns when giving anti-diabetes, therapeutic decisions needed to be made with caution when diabetic patients were already at a high risk of fractures. Our conclusion may help for the proper prescription for patients in different condition. In addition, further studies are needed to clarify pre-existing paradox.

## Supporting information

S1 File PRISMA NMA checking list. (DOCX)

S1 Table Quality assessment of included trials by Cochrane risk of bias tool. Note: GLP-1RAs: (Glucagon-like peptide-1) receptor agonists; SGLT-2: Sodium-Glucose co-Transporter 2; Met: metformin; SU: sulphanylureas; TZD: thiazolidinediones.
(DOCX)

S2 Table Node split method for inconsistence check in network. Note: GLP-1RAs: (Glucagon-like peptide-1) receptor agonists; SGLT-2: Sodium-Glucose co-Transporter 2; Met: metformin; SU: sulphanylureas; TZD: thiazolidinediones.
(DOCX)

S3 Table Odds ratio (OR) with 95% confidence interval (CI) of NMA for bone fracture events based on a frequentist framework when compared with placebo. Note: NA: not available. GLP-1RAs (Glucagon-like peptide-1) receptor agonists; SGLT-2: Sodium-Glucose co-Transporter 2; Met: metformin; SU: sulphanylureas; TZDs: thiazolidinediones.
(DOCX)

## References

[1] Fadini GP, Bonora BM, Cappellari R, Menegazzo L, Vedovato M, Iori E, et al Acute Effects of Linagliptin on Progenitor Cells, Monocyte Phenotypes, and Soluble Mediators in Type 2 Diabetes. J Clin Endocrinol Metab. 2016;101(2):748–56. doi: 10.1210/jc.2015-3716 26695864 10.1210/jc.2015-3716

[2] Godoy-Matos AF. The role of glucagon on type 2 diabetes at a glance. Diabetol Metab Syndr. 2014;6(1):91 doi: 10.1186/1758-5996-6-91 25177371 10.1186/1758-5996-6-91 PMC4148933

[3] Fan Y, Wei F, Lang Y, Liu Y. Diabetes mellitus and risk of hip fractures: a meta-analysis. Osteoporos Int. 2016;27(1):219–28. doi: 10.1007/s00198-015-3279-7 26264604 10.1007/s00198-015-3279-7

[4] Janghorbani M, Van Dam RM, Willett WC, Hu FB. Systematic Review of Type 1 and Type 2 Diabetes Mellitus and Risk of Fracture. Am J Epidemiol. 2007;166(5):495–505. doi: 10.1093/aje/kwm106 17575306 10.1093/aje/kwm106

[5] Ceccarelli E, Guarino EG, Merlotti D, Patti A, Gennari L, Nuti R, et al Beyond Glycemic Control in Diabetes Mellitus: Effects of Incretin-Based Therapies on Bone Metabolism. Front Endocrinol (Lausanne). 2013;4:73 doi: 10.3389/fendo.2013.00073 23785355 10.3389/fendo.2013.00073 PMC3684850

[6] Yamaguchi T, Sugimoto T. Bone metabolism and fracture risk in type 2 diabetes mellitus. Bonekey Rep. 2012;1:36 doi: 10.1038/bonekey.2012.27 23951442 10.1038/bonekey.2012.27 PMC3727726

[7] Yabe D, Seino Y. Dipeptidyl peptidase-4 inhibitors and prevention of bone fractures: Effects beyond glyemic control. J Diabetes Investig. 2012;3(4):347–8. doi: 10.1111/j.2040-1124.2012.00219.x 24843587 10.1111/j.2040-1124.2012.00219.x PMC4019252

[8] Conti F, Wolosinska DT, Pugliese G. Diabetes and bone fragility: a dangerous liaison. Aging Clin Exp Res. 2013;25 Suppl 1:S39–41. doi: 10.1007/s40520-013-0084-z 23907773 10.1007/s40520-013-0084-z

[9] Billington EO, Grey A, Bolland MJ. The effect of thiazolidinediones on bone mineral density and bone turnover: systematic review and meta-analysis. Diabetologia. 2015;58(10):2238–46. doi: 10.1007/s00125-015-3660-2 26109213 10.1007/s00125-015-3660-2

[10] Pratley RE, Salsali A. Inhibition of DPP-4: a new therapeutic approach for the treatment of type 2 diabetes. Curr Med Res Opin. 2007;23(4):919–31. doi: 10.1185/030079906X162746 17407649 10.1185/030079906x162746

[11] Scheen AJ. Pharmacokinetics of dipeptidylpeptidase-4 inhibitors. Diabetes Obes Metab. 2010;12(8):648–58. doi: 10.1111/j.1463-1326.2010.01212.x 20590741 10.1111/j.1463-1326.2010.01212.x

[12] Barchetta I, Cimini FA, Bloise D, Cavallo MG. Dipeptidyl peptidase-4 inhibitors and bone metabolism: is vitamin D the link? Acta Diabetol. 2016;53(5):839–44. doi: 10.1007/s00592-016-0882-9 27379733 10.1007/s00592-016-0882-9

[13] Bunck MC, Poelma M, Eekhoff EM, Schweizer A, Heine RJ, Nijpels G, et al Effects of vildagliptin on postprandial markers of bone resorption and calcium homeostasis in recently diagnosed, well-controlled type 2 diabetes patients. J Diabetes. 2012;4(2):181–5. doi: 10.1111/j.1753-0407.2011.00168.x 22051153 10.1111/j.1753-0407.2011.00168.x

[14] Mosenzon O, Wei C, Davidson J, Scirica BM, Yanuv I, Rozenberg A, et al Incidence of Fractures in Patients With Type 2 Diabetes in the SAVOR-TIMI 53 Trial. Diabetes Care. 2015;38(11):2142–50. doi: 10.2337/dc15-1068 26358285 10.2337/dc15-1068

[15] Driessen JH, van Onzenoort HA, Henry RM, Lalmohamed A, van den Bergh JP, Neef C, et al Use of dipeptidyl peptidase-4 inhibitors for type 2 diabetes mellitus and risk of fracture. Bone. 2014;68:124–30. doi: 10.1016/j.bone.2014.07.030 25093264 10.1016/j.bone.2014.07.030

[16] Hirshberg B, Parker A, Edelberg H, Donovan M, Iqbal N. Safety of saxagliptin: events of special interest in 9156 patients with type 2 diabetes mellitus. Diabetes Metab Res Rev. 2014;30(7):556–69. doi: 10.1002/dmrr.2502 24376173 10.1002/dmrr.2502

[17] Mamza J, Marlin C, Wang C, Chokkalingam K, Idris I. DPP-4 inhibitor therapy and bone fractures in people with Type 2 diabetes—A systematic review and meta-analysis. Diabetes Res Clin Pract. 2016;116:288–98. doi: 10.1016/j.diabres.2016.04.029 27321347 10.1016/j.diabres.2016.04.029

[18] Monami M, Dicembrini I, Antenore A, Mannucci E. Dipeptidyl peptidase-4 inhibitors and bone fractures: a meta-analysis of randomized clinical trials. Diabetes Care. 2011;34(11):2474–6. doi: 10.2337/dc11-1099 22025784 10.2337/dc11-1099 PMC3198283

[19] Rucker G, Schwarzer G, Carpenter J, Olkin I. Why add anything to nothing? The arcsine difference as a measure of treatment effect in meta-analysis with zero cells. Stat Med. 2009;28(5):721–38. doi: 10.1002/sim.3511 19072749 10.1002/sim.3511

[20] van Valkenhoef G, Lu G, de Brock B, Hillege H, Ades AE, Welton NJ. Automating network meta-analysis. Res Synth Methods. 2012;3(4):285–99. doi: 10.1002/jrsm.1054 26053422 10.1002/jrsm.1054

[21] Higgins J, G S E. Cochrane Handbook for Systematic Reviews of Interventions Version 5. 1. 0: Available at: http://www.cochrane-handbook.org %\2016-08-25 11:35:00.

[22] DerSimonian R, Kacker R. Random-effects model for meta-analysis of clinical trials: an update. Contemp Clin Trials. 2007 2;28(2):105–14. doi: 10.1016/j.cct.2006.04.004 16807131 10.1016/j.cct.2006.04.004

[23] Higgins JP, Thompson SG. Quantifying heterogeneity in a meta-analysis. Stat Med. 2002;21(11):1539–58. doi: 10.1002/sim.1186 12111919 10.1002/sim.1186

[24] Higgins JP, Thompson SG, Deeks JJ, Altman DG. Measuring inconsistency in meta-analyses. BMJ. 2003;327(7414):557–60. doi: 10.1136/bmj.327.7414.557 12958120 10.1136/bmj.327.7414.557 PMC192859

[25] Salanti G. Indirect and mixed-treatment comparison, network, or multiple-treatments meta-analysis: many names, many benefits, many concerns for the next generation evidence synthesis tool. Res Synth Methods. 2012;3(2):80–97. doi: 10.1002/jrsm.1037 26062083 10.1002/jrsm.1037

[26] Song F, Altman DG, Glenny AM, Deeks JJ. Validity of indirect comparison for estimating efficacy of competing interventions: empirical evidence from published meta-analyses. BMJ. 2003;326(7387):472 doi: 10.1136/bmj.326.7387.472 12609941 10.1136/bmj.326.7387.472 PMC150178

[27] van Valkenhoef G, Dias S, Ades AE, Welton NJ. Automated generation of node-splitting models for assessment of inconsistency in network meta-analysis. Res Synth Methods. 2016;7(1):80–93. doi: 10.1002/jrsm.1167 26461181 10.1002/jrsm.1167 PMC5057346

[28] Riley RD, Higgins JPT, Deeks JJ. Interpretation of random effects meta-analyses. BMJ. 2011;342:d549 doi: 10.1136/bmj.d549 21310794 10.1136/bmj.d549

[29] Egger M, Davey SG, Schneider M, Minder C. Bias in meta-analysis detected by a simple, graphical test. BMJ. 1997;315(7109):629–34. 9310563 10.1136/bmj.315.7109.629 PMC2127453

[30] White IR, Barrett JK, Jackson D, Higgins JPT. Consistency and inconsistency in network meta-analysis: model estimation using multivariate meta-regression. Res Synth Methods. 2012;3(2):111–25. doi: 10.1002/jrsm.1045 26062085 10.1002/jrsm.1045 PMC4433771

[31] Dede AD, Tournis S, Dontas I, Trovas G. Type 2 diabetes mellitus and fracture risk. Metabolism. 2014;63(12):1480–90. doi: 10.1016/j.metabol.2014.09.002 25284729 10.1016/j.metabol.2014.09.002

[32] Giangregorio LM, Leslie WD, Lix LM, Johansson H, Oden A, McCloskey E, et al FRAX underestimates fracture risk in patients with diabetes. J Bone Miner Res. 2012;27(2):301–8. doi: 10.1002/jbmr.556 22052532 10.1002/jbmr.556

[33] Strotmeyer ES, Kamineni A, Cauley JA, Robbins JA, Fried LF, Siscovick DS, et al Potential Explanatory Factors for Higher Incident Hip Fracture Risk in Older Diabetic Adults. Curr Gerontol Geriatr Res. 2011;2011:979270 doi: 10.1155/2011/979270 21837239 10.1155/2011/979270 PMC3152969

[34] Yabe D, Seino Y. Alogliptin for the treatment of type 2 diabetes: a drug safety evaluation. Expert Opin Drug Saf. 2016;15(2):249–64. doi: 10.1517/14740338.2016.1125467 26607297 10.1517/14740338.2016.1125467

[35] Fu J, Zhu J, Hao Y, Guo C, Zhou Z. Dipeptidyl peptidase-4 inhibitors and fracture risk: an updated meta-analysis of randomized clinical trials. Sci Rep. 2016;6:29104 doi: 10.1038/srep29104 27384445 10.1038/srep29104 PMC4935882

[36] Mannucci E, Dicembrini I. Drugs for type 2 diabetes: role in the regulation of bone metabolism. Clin Cases Miner Bone Metab. 2015;12(2):130–4. doi: 10.11138/ccmbm/2015.12.2.130 26604937 10.11138/ccmbm/2015.12.2.130 PMC4625768

[37] Glorie L, Behets GJ, Baerts L, De Meester I, D'Haese PC, Verhulst A. DPP IV inhibitor treatment attenuates bone loss and improves mechanical bone strength in male diabetic rats. Am J Physiol Endocrinol Metab. 2014;307(5):E447–55. doi: 10.1152/ajpendo.00217.2014 25053403 10.1152/ajpendo.00217.2014

[38] Scirica BM. Alogliptin after Acute Coronary Syndrome in Patients with Type 2 Diabetes. N Engl J Med. 2013;369(14):1327–35. doi: 10.1056/NEJMoa1305889 23992602 10.1056/NEJMoa1305889

[39] Shah Z, Kampfrath T, Deiuliis JA, Zhong J, Pineda C, Ying Z, et al Chronic DPP-4 Inhibition Reduces Atherosclerosis and Inflammation via Effects on Monocyte Recruitment and Chemotaxis. Circulation. 2011;124(21):2338–49. doi: 10.1161/CIRCULATIONAHA.111.041418 22007077 10.1161/CIRCULATIONAHA.111.041418 PMC4224594

[40] Kania DS, Gonzalvo JD, Weber ZA. Saxagliptin: A Clinical Review in the Treatment of Type 2 Diabetes Mellitus. Clin Ther. 2011;33(8):1005–22. doi: 10.1016/j.clinthera.2011.06.016 21802144 10.1016/j.clinthera.2011.06.016

[41] Evans PMS, Bain SC. Omarigliptin for the treatment of Type 2 diabetes mellitus. Expert Opin Pharmacother. 2016;17(14):1947–52. doi: 10.1080/14656566.2016.1218472 27466703 10.1080/14656566.2016.1218472

[42] Hermansen K, Kipnes M, Luo E, Fanurik D, Khatami H, Stein P, et al Efficacy and safety of the dipeptidyl peptidase-4 inhibitor, sitagliptin, in patients with type 2 diabetes mellitus inadequately controlled on glimepiride alone or on glimepiride and metformin. Diabetes Obes Metab. 2007;9(5):733–45. doi: 10.1111/j.1463-1326.2007.00744.x 17593236 10.1111/j.1463-1326.2007.00744.x

[43] Wang MC, Bachrach LK, Van Loan M, Hudes M, Flegal KM, Crawford PB. The relative contributions of lean tissue mass and fat mass to bone density in young women. Bone. 2005;37(4):474–81. doi: 10.1016/j.bone.2005.04.038 16040285 10.1016/j.bone.2005.04.038

[44] Zinman B, Haffner SM, Herman WH, Holman RR, Lachin JM, Kravitz BG, et al Effect of rosiglitazone, metformin, and glyburide on bone biomarkers in patients with type 2 diabetes. J Clin Endocrinol Metab. 2010;95(1):134–42. doi: 10.1210/jc.2009-0572 19875477 10.1210/jc.2009-0572

[45] Lapane KL, Yang S, Brown MJ, Jawahar R, Pagliasotti C, Rajpathak S. Sulfonylureas and risk of falls and fractures: a systematic review. Drugs Aging. 2013;30(7):527–47. doi: 10.1007/s40266-013-0081-0 23609875 10.1007/s40266-013-0081-0

[46] Lapane KL, Jesdale BM, Dube CE, Pimentel CB, Rajpathak SN. Sulfonylureas and risk of falls and fractures among nursing home residents with type 2 diabetes mellitus. Diabetes Res Clin Pract. 2015;109(2):411–9. doi: 10.1016/j.diabres.2015.05.009 26008723 10.1016/j.diabres.2015.05.009

[47] Ma P, Gu B, Ma J, E L, Wu X, Cao J, et al Glimepiride induces proliferation and differentiation of rat osteoblasts via the PI3-kinase/Akt pathway. Metabolism. 2010;59(3):359–66. doi: 10.1016/j.metabol.2009.08.003 19800638 10.1016/j.metabol.2009.08.003

[48] Fronczek-Sokol J, Pytlik M. Effect of glimepiride on the skeletal system of ovariectomized and non-ovariectomized rats. Pharmacol Rep. 2014;66(3):412–7. doi: 10.1016/j.pharep.2013.12.013 24905517 10.1016/j.pharep.2013.12.013

[49] Driessen JHM, Henry RMA, van Onzenoort HAW, Lalmohamed A, Burden AM, Prieto-Alhambra D, et al Bone Fracture Risk is Not Associated with the Use of Glucagon-Like Peptide-1 Receptor Agonists: A Population-Based Cohort Analysis. Calcif Tissue Int. 2015;97(2):104–12. doi: 10.1007/s00223-015-9993-5 25894068 10.1007/s00223-015-9993-5 PMC4491344

[50] Mabilleau G, Mieczkowska A, Chappard D. Use of glucagon-like peptide-1 receptor agonists and bone fractures: a meta-analysis of randomized clinical trials. J Diabetes. 2014;6(3):260–6. doi: 10.1111/1753-0407.12102 24164867 10.1111/1753-0407.12102

[51] Su B, Sheng H, Zhang M, Bu L, Yang P, Li L, et al Risk of bone fractures associated with glucagon-like peptide-1 receptor agonists’ treatment: a meta-analysis of randomized controlled trials. Endocrine. 2015;48(1):107–15. doi: 10.1007/s12020-014-0361-4 25074632 10.1007/s12020-014-0361-4

[52] Driessen JHM, van Onzenoort HAW, Starup-Linde J, Henry R, Burden AM, Neef C, et al Use of Glucagon-Like-Peptide 1 Receptor Agonists and Risk of Fracture as Compared to Use of Other Anti-hyperglycemic Drugs. Calcif Tissue Int. 2015;97(5):506–15. doi: 10.1007/s00223-015-0037-y 26184119 10.1007/s00223-015-0037-y PMC4598352

[53] Kawanami D, Matoba K, Sango K, Utsunomiya K. Incretin-Based Therapies for Diabetic Complications: Basic Mechanisms and Clinical Evidence. Int J Mol Sci. 2016;17(8). pii: E1223 doi: 10.3390/ijms17081223 27483245 10.3390/ijms17081223 PMC5000621

[54] Zhong Q, Itokawa T, Sridhar S, Ding KH, Xie D, Kang B, et al Effects of glucose-dependent insulinotropic peptide on osteoclast function. Am J Physiol Endocrinol Metab. 2007;292(2):E543–8. doi: 10.1152/ajpendo.00364.2006 17003233 10.1152/ajpendo.00364.2006

[55] Xie D, Cheng H, Hamrick M, Zhong Q, Ding KH, Correa D, et al Glucose-dependent insulinotropic polypeptide receptor knockout mice have altered bone turnover. Bone. 2005;37(6):759–69. doi: 10.1016/j.bone.2005.06.021 16219496 10.1016/j.bone.2005.06.021

[56] Driessen JH, de Vries F, van Onzenoort H, Harvey NC, Neef C, van den Bergh JP, et al The use of incretins and fractures—a meta-analysis on population-based real life data. Br J Clin Pharmacol. 2017;83(4):923–926. doi: 10.1111/bcp.13167 27780288 10.1111/bcp.13167 PMC5346876

