# Supplementary material for: The effects of dipeptidyl peptidase-4 inhibitors on bone fracture among patients with type 2 diabetes mellitus: A network meta-analysis of randomized controlled trials
Source: PLoS One. 2017 Dec 5;12(12):e0187537. doi: 10.1371/journal.pone.0187537 (PMC5716604; doi:10.1371/journal.pone.0187537)
Supplement: S1 Table — Note: GLP-1RAs: (Glucagon-like peptide-1) receptor agonists; SGLT-2: Sodium-Glucose co-Transporter 2; Met: metformin; SU: sulphanylureas; TZD: thiazolidinediones. (DOCX) [file pone.0187537.s002.docx]

**S1 Table. Quality assessment of included trials by Cochrane risk of bias tool**

| **Study** | **Registry No.** | **Random Sequence generation** | **Allocation concealment** | **Blinding of participants &personnel** | **Blinding of outcome assessment** | **Incomplete outcome data** | **Selective reporting** | **Company funding** |
| --- | --- | --- | --- | --- | --- | --- | --- | --- |
| Arjona Ferreira JC 2013[[1](#_ENREF_1)A] | NCT00509262 | low | low | low | unclear | low | low | Y |
| Arjona Ferreira JC 2013[[2](#_ENREF_2)A] | NCT00509236 | low | low | low | low | low | low | Y |
| Aschner P 2010[[3](#_ENREF_3)A] | NCT00449930 | low | low | low | unclear | low | low | Y |
| Barnett AH 2013[4A] | NCT00757588 | low | low | low | unclear | low | low | Y |
| Barnett AH 2013[5A] | NCT00757588 | low | low | low | unclear | low | low | Y |
| Barnett AH 2013[6A] | NCT01084005 | low | low | low | low | low | low | Y |
| Barzilai N 2011[7A] | NCT00305604 | low | low | high | unclear | low | low | Y |
| CANTATA-D, Lavalle-Gonzalez FJ 2013[8A] | NCT01106677 | low | low | low | unclear | low | low | Y |
| CANTATA-D2, Schernthaner G 2013[9A] | NCT01137812 | low | low | low | low | low | low | Y |
| Charbonnel B 2006[10A] | NCT00086515 | low | low | low | unclear | low | low | Y |
| DeFronzo RA 2012[11A] | NCT00328627 | low | low | low | unclear | low | low | Y |
| Dobs AS 2013[12A] | NCT00350779 | low | low | high | low | low | low | Y |
| ENDURE, NCT00856284 2013[13A] | NCT00856284 | low | unclear | low | unclear | low | low | Y |
| Fonseca V 2013[[14](#_ENREF_34)A] | NCT00885352 | low | low | low | unclear | low | low | Y |
| Frederich R 2012[15A] | NCT00316082 | low | low | low | unclear | low | low | Y |
| Göke B 2013[16A] | NCT00575588 | low | low | low | low | low | low | Y |
| Haak T 2013[17A] | NCT00915772 | low | low | low | unclear | low | low | Y |
| Jadzinsky M 2009[[18](#_ENREF_48)A] | NCT00327015 | low | low | low | unclear | low | low | Y |
| Kashiwagi A 2011[19A] | NCT00372060 | low | low | low | unclear | low | low | Y |
| NCT00121667 2014[20A] | NCT00121667 | low | low | low | unclear | low | low | Y |
| Raz I 2008[21A] | NCT00337610 | low | low | low | unclear | low | low | Y |
| NCT00601250 2009[22A] | NCT00601250 | low | low | low | unclear | low | low | Y |
| NCT00602472 2014[23A] | NCT00602472 | low | low | low | unclear | low | low | Y |
| NCT00661362 2012[24A] | NCT00661362 | low | low | low | low | low | low | Y |
| Arechavaleta R 2011[25A] | NCT00701090 | low | low | low | unclear | low | low | Y |
| Henry RR 2014[26A] | NCT00722371 | low | low | low | low | low | low | Y |
| NCT00798161 2010[27A] | NCT00798161 | low | low | low | unclear | low | low | Y |
| Sheu WHH 2015[28A] | NCT00954447 | low | low | low | low | low | low | Y |
| GENERATION, Schernthaner G 2015[29A] | NCT01006603 | low | unclear | low | unclear | low | low | Y |
| Yoon KH 2012[30A] | NCT00397631 | low | low | low | low | low | low | Y |
| NCT01076088 2015[31A] | NCT01076088 | low | unclear | low | unclear | low | low | Y |
| NCT01183013 2014[32A] | NCT01183013 | low | unclear | low | unclear | low | low | Y |
| NCT01204294 2012[33A] | NCT01204294 | low | unclear | high | unclear | low | low | Y |
| NCT01215097 2013[34A] | NCT01215097 | low | unclear | low | unclear | low | low | Y |
| Ji L 2015[[35](#_ENREF_95)A] | NCT01215097 | low | low | low | unclear | low | low | Y |
| Mathieu C 2015[[36](#_ENREF_96)A] | NCT01462266 | low | low | low | unclear | low | low | Y |
| Nauck MA 2007[37A] | NCT00094770 | low | low | low | unclear | low | low | Y |
| Nowicki M 2011[[38](#_ENREF_102)A] | NCT00614939 | low | low | low | unclear | low | low | Y |
| Olansky L 2011[[39](#_ENREF_103)A] | NCT00482729 | low | low | low | unclear | low | low | Y |
| Pratley RE 2009[[40](#_ENREF_110)A] | NCT00286468 | low | low | low | low | low | low | Y |
| Prato S 2011[[41](#_ENREF_112)A] | NCT00621140 | low | low | low | unclear | low | low | Y |
| Raz I 2006[[42](#_ENREF_113)A] | NCT00087516 | low | low | low | low | low | low | Y |
| Roden M 2015[[43](#_ENREF_114)A] | NCT01289990 | low | low | low | unclear | low | low | Y |
| Rosenstock J 2006[[44](#_ENREF_115)A] | NCT00086502 | low | low | low | unclear | low | low | Y |
| Rosenstock J 2009[[45](#_ENREF_121)A] | NCT00121641 | low | low | low | unclear | low | low | Y |
| Rosenstock J 2013[[46](#_ENREF_122)A] | NCT00707993 | low | low | low | low | low | low | Y |
| Ross SA 2012[[47](#_ENREF_123)A] | NCT01012037 | low | low | low | low | low | low | Y |
| SAVOR-TIMI 53, Raz I 2014[[48](#_ENREF_124)A] | NCT01107886 | low | low | low | low | low | low | Y |
| Vilsboll T 2010[[49](#_ENREF_134)A] | NCT00395343 | low | low | low | low | low | low | Y |
| Wainstein J 2012[[50](#_ENREF_136)A] | NCT00532935 | low | low | low | low | low | low | Y |
| EXAMINE, White WB 2013[[51](#_ENREF_137)A] | NCT00968708 | low | low | low | low | low | low | Y |
| Goldstein BJ 2007[[52](#_ENREF_138)A] | NCT00103857 | low | low | high | low | low | low | Y |
| Pratley RE 2014[[53](#_ENREF_140)A] | NCT01023581 | low | low | low | low | low | low | Y |
| Takihata M 2013[[54](#_ENREF_144)A] | UMIN000004716 | low | low | high | high | low | low | N |
| Gallwitz B 2012[[55](#_ENREF_153)A] | NCT00622284 | low | low | low | low | low | low | Y |
| Rosenstock J 2015[[56](#_ENREF_179)A] | NCT01606007 | low | low | low | unclear | low | low | Y |
| Hirose T 2015[[57](#_ENREF_181)A] | NCT02002221 | low | low | low | low | low | low | Y |
| Mita T 2016[[58](#_ENREF_186)A] | UMIN000007396 | low | low | high | high | low | low | N |
| Hollander PL 2011[59A] | NCT00295633 | low | low | high | unclear | low | low | Y |
| Charbonnel B 2013[60A] | NCT01296412 | low | low | high | unclear | low | low | Y |
| Nauck M 2007[61A] | NCT00094770 | low | low | low | NR | NR | NR | NR |
| AWARD-5, Weinstock RS 2015[62A] | NCT00734474 | low | low | low | low | low | low | Y |
| Roden M 2013[63A] | NCT01177813 | low | low | low | low | low | low | Y |
| Takeshita Y 2015[64A] | UMIN000004953 | low | low | high | high | low | low | N |
| NCT01098539 2014[65A] | NCT01098539 | low | unclear | low | unclear | low | low | Y |
| Moses RG 2015[66A]  NCT01545388 2014[67A] | NCT01076075  NCT01545388 | low  low | low  low | low  low | low  low | NR  low | NR  low | NR  Y |
| HAMORNY3 2014[68A] | NCT00838903 | low | low | low | low | low | low | Y |
| Bosi E 2011[69A] | NCT00432276 | low | low | low | low | low | low | Y |
| Bosi E 2009[70A] | NCT00468039 | low | unclear | low | low | low | low | Y |
| Fonseca 2007[71A] | NCT00099931 | low | low | low | unclear | low | low | Y |
| Pan C 2012[72A] | NR | low | unclear | low | unclear | low | low | Y |
| Scherbaum WA 2008[73A] | NCT00101712 | low | low | low | unclear | low | low | Y |
| McGill JB 2014[74A] | NCT00800683 | low | low | low | low | low | low | Y |
| Ferrannini E2013[75A] | NCT00881530 | low | unclear | high | unclear | low | low | Y |

Note: NR: not report; Y: yes; N: no; CANTATA-D Trial: CANagliflozin Treatment and Trial Analysis-DPP-4 Inhibitor Comparator Trial; CANTATA-D2: CANagliflozin Treatment And Trial Analysis-DPP-4 Inhibitor Second Comparator Trial; ENDURE: Efficacy and Safety of Alogliptin Plus Metformin Compared to Glipizide Plus Metformin in Patients With Type 2 Diabetes Mellitus; GENERATION: Saxagliptin Compared to Glimepiride in Elderly Type 2 Diabetes Patients, With Inadequate Glycemic Control on Metformin; EXAMINE: Examination of Cardiovascular Outcomes with Alogliptin versus Standard of Care; ELIXA: Evaluation of Lixisenatide in Acute Coronary Syndrome; AWARD-5: Safety and efficacy of once-weekly dulaglutide versus sitagliptin after 2 years in metformin-treated patients with type 2 diabetes

1A. Arjona Ferreira, J.C., et al., *Efficacy and safety of sitagliptin versus glipizide in patients with type 2 diabetes andmoderate-to-severe chronic renal insufficiency.* Diabetes Care, 2013. **36**(5): p. 1067-1073.

2A. Arjona Ferreira, J.C., et al., *Efficacy and safety of sitagliptin in patients with type 2 diabetes and ESRD receiving dialysis: A 54-week randomized trial.* American Journal of Kidney Diseases, 2013. **61**(4): p. 579-587.

3A. Aschner, P., et al., *Efficacy and safety of monotherapy of sitagliptin compared with metformin in patients with type 2 diabetes.* Diabetes, Obesity and Metabolism, 2010. **12**(3): p. 252-261.

4A. Barnett, A.H., et al., *Saxagliptin add-on therapy to insulin with or without metformin for type 2 diabetes mellitus: 52-week safety and efficacy.* Clin Drug Investig, 2013. **33**(10): p. 707-17.

5A. Barnett, A.H., et al., *Effect of saxagliptin as add-on therapy in patients with poorly controlled type 2 diabetes on insulin alone or insulin combined with metformin.* Curr Med Res Opin. 2012, **28**(4):p. 513-523.

6A. Barnett, A.H., et al., *Linagliptin for patients aged 70 years or older with type 2 diabetes inadequately controlled with common antidiabetes treatments: a randomised, double-blind, placebo-controlled trial.* Lancet, 2013. **382**(9902): p. 1413-23.

7A. Barzilai, N., et al., *Efficacy and tolerability of sitagliptin monotherapy in elderly patients with type 2 diabetes: A randomized, double-blind, placebo-controlled trial.* Current Medical Research and Opinion, 2011. **27**(5): p. 1049-1058.

8A. Lavalle-Gonzalez, F.J., et al., *Efficacy and safety of canagliflozin compared with placebo and sitagliptin in patients with type 2 diabetes on background metformin monotherapy: A randomised trial.* Diabetologia, 2013. **56**(12): p. 2582-2592.

9A. Schernthaner, G., et al., *Canagliflozin compared with sitagliptin for patients with type 2 diabetes who do not have adequate glycemic control with metformin plus sulfonylurea: A 52-week randomized trial.* Diabetes Care, 2013. **36**(9): p. 2508-2515.

10A. Charbonnel, B., et al., *Efficacy and safety of the dipeptidyl peptidase-4 inhibitor sitagliptin added to ongoing metformin therapy in patients with type 2 diabetes inadequately controlled with metformin alone.* Diabetes Care, 2006. **29**(12): p. 2638-2643.

11A. DeFronzo, R.A., et al., *Efficacy and tolerability of the DPP-4 inhibitor alogliptin combined with pioglitazone, in metformin-treated patients with type 2 diabetes.* J Clin Endocrinol Metab, 2012. **97**(5): p. 1615-22.

12A. Dobs, A.S., et al., *Efficacy and safety of sitagliptin added to ongoing metformin and rosiglitazone combination therapy in a randomized placebo-controlled 54-week trial in patients with type 2 diabetes.* Journal of Diabetes, 2013. **5**(1): p. 68-79.

13A. *Efficacy and Safety of Alogliptin Plus Metformin Compared to Glipizide Plus Metformin in Patients With Type 2 Diabetes Mellitus.* <http://ClinicalTrials.gov/show/NCT00856284>.

14A. Fonseca, V., et al., *Efficacy and safety of sitagliptin added to ongoing metformin and pioglitazone combination therapy in a randomized, placebo-controlled, 26-week trial in patients with type 2 diabetes.* J Diabetes Complications, 2013. **27**(2): p. 177-83.

15A. Frederich, R., et al., *The efficacy and safety of the dipeptidyl peptidase-4 inhibitor saxagliptin in treatment-naive patients with type 2 diabetes mellitus: a randomized controlled trial.* Diabetol Metab Syndr, 2012. **4**(1): p. 36.

16A. Göke, B., et al. (2013) *Saxagliptin vs. glipizide as add-on therapy in patients with type 2 diabetes mellitus inadequately controlled on metformin alone: long-term (52-week) extension of a 52-week randomised controlled trial*. International journal of clinical practice **67**, 307-16.

17A. Haak, T., et al., *Initial combination of linagliptin and metformin in patients with type 2 diabetes: efficacy and safety in a randomised, double-blind 1-year extension study.* Int J Clin Pract, 2013. **67**(12): p. 1283-93.

18A. Jadzinsky, M., et al., *Saxagliptin given in combination with metformin as initial therapy improves glycaemic control in patients with type 2 diabetes compared with either monotherapy: a randomized controlled trial.* Diabetes Obes Metab, 2009. **11**(6): p. 611-22.

19A. Kashiwagi, A., et al., *Sitagliptin added to treatment with ongoing pioglitazone for up to 52 weeks improves glycemic control in Japanese patients with type 2 diabetes.* Journal of Diabetes Investigation, 2011. **2**(5): p. 381-390.

20A. *Study Assessing Saxagliptin Treatment In Type 2 Diabetic Subjects Who Are Not Controlled With Metformin Alone.* Has Results.

21A. Raz, I., et al., *Efficacy and safety of sitagliptin added to ongoing metformin therapy in patients with type 2 diabetes.* Current Medical Research and Opinion, 2008. **24**(2): p. 537-550.

22A. *Efficacy and Safety of B I1356 (Linagliptin) vs. Placebo Added to Metformin Background Therapy in Patients With Type 2 Diabetes* [*http://ClinicalTrials.gov/show/NCT00601250*](http://ClinicalTrials.gov/show/NCT00601250)*.* Has Results.

23A. *BI 1356 (Linagliptin) in Combination With Metformin and a Sulphonylurea in Type 2 Diabetes.* Has Results.

24A. *Evaluate Efficacy and Safety of Saxagliptin in Combination With Metformin in Adult Patients With Type 2 Diabetes.* Has Results.

25A. Arechavaleta, R., et al. (2011) *Efficacy and safety of treatment with sitagliptin or glimepiride in patients with type 2 diabetes inadequately controlled on metformin monotherapy: a randomized, double-blind, non-inferiority trial*. Diabetes, obesity & metabolism **13**, 160-8 DOI: 10.1111/j.1463-1326.2010.01334.x.

26A. Henry RR, S.B., Fonseca VA, Chou MZ, Teng R, Golm GT, Langdon RB, Kaufman KD, Steinberg H, Goldstein BJ., *Efficacy and safety of initial combination treatment with sitagliptin and pioglitazone--a factorial study.* Diabetes Obes Metab., 2014. **2014 Mar;16(3):223-30.**

27A. Boehringer Ingelheim, P., *Safety and Efficacy of Linagliptin (BI 1356) Plus Metformin in Type 2 Diabetes, Factorial Design*. 2010.

28A. Sheu, W.H.H., et al., *Linagliptin improves glycemic control after 1 year as add-on therapy to basal insulin in Asian patients with type 2 diabetes mellitus.* Current medical research and opinion, 2015. **31 (3)**: p. 503-512.

29A. Schernthaner, G., et al., *Efficacy and tolerability of saxagliptin compared with glimepiride in elderly patients with type 2 diabetes: A randomized, controlled study (GENERATION).* Diabetes, Obesity and Metabolism, 2015. **17 (7)**: p. 630-638.

30A. Yoon, K.H., et al., *Efficacy and safety of initial combination therapy with sitagliptin and pioglitazone in patients with type 2 diabetes: a 54-week study.* Diabetes Obes Metab, 2012. **14**(8): p. 745-52.

31A. *Safety and Efficacy of Co-Administration of Sitagliptin and Metformin in China (MK-0431-121).* Has Results.

32A. *30 Week Parallel Group Comparison Study of Linagliptin + Pioglitazone (5+15, 5+30 and 5+45 mg) qd Versus Respective Monotherapies, Followed by a Comparison of 5mg+30mg and 5mg+45mg Versus Respective Monotherapies in Type 2 Diabetes for up to 54 Weeks.* <http://ClinicalTrials.gov/show/NCT01183013>.

33A. Boehringer Ingelheim, P., L. Eli, and Company, *Comprehensive Add on Study in Japan*. 2012.

34A. *Efficacy and Safety Study of Linagliptin (5 mg Administered Orally Once Daily) Over 24 Weeks in Type 2 Diabetic Patients With Insufficient Glycaemic Control Despite Metformin Therapy.* Has Results.

35A. Ji, L., et al., *Efficacy and Safety of Linagliptin Co-Administered with Low-Dose Metformin Once Daily Versus High-Dose Metformin Twice Daily in Treatment-Naïve Patients with Type 2 Diabetes: a Double-Blind Randomized Trial.* Advances in Therapy, 2015. **32**(3): p. 201-215.

36A. Mathieu, C., et al., *A Randomized Clinical Trial to Evaluate the Efficacy and Safety of Co-Administration of Sitagliptin with Intensively Titrated Insulin Glargine.* Diabetes Therapy, 2015. **6 (2)**: p. 127-142.

37A. Nauck, M.A., et al., *Efficacy and safety of the dipeptidyl peptidase-4 inhibitor, sitagliptin, compared with the sulfonylurea, glipizide, in patients with type 2 diabetes inadequately controlled on metformin alone: A randomized, double-blind, non-inferiority trial.* Diabetes, Obesity and Metabolism, 2007. **9**(2): p. 194-205.

38A. Nowicki, M., et al., *Saxagliptin improves glycaemic control and is well tolerated in patients with type 2 diabetes mellitus and renal impairment.* Diabetes Obes Metab, 2011. **13**(6): p. 523-32.

39A. Olansky, L., et al., *A treatment strategy implementing combination therapy with sitagliptin and metformin results in superior glycaemic control versus metformin monotherapy due to a low rate of addition of antihyperglycaemic agents.* Diabetes, Obesity and Metabolism, 2011. **13**(9): p. 841-849.

40A. Pratley, R.E., et al., *Efficacy and safety of the dipeptidyl peptidase-4 inhibitor alogliptin in patients with type 2 diabetes inadequately controlled by glyburide monotherapy.* Diabetes, Obesity and Metabolism, 2009. **11**(2): p. 167-176.

41A. Prato, S., et al. (2011) *Effect of linagliptin monotherapy on glycaemic control and markers of ?-cell function in patients with inadequately controlled type 2 diabetes: a randomized controlled trial*. Diabetes, obesity & metabolism **13**, 258-67 DOI: 10.1111/j.1463-1326.2010.01350.x.

42A. Raz, I., et al., *Efficacy and safety of the dipeptidyl peptidase-4 inhibitor sitagliptin as monotherapy in patients with type 2 diabetes mellitus.* Diabetologia, 2006. **49**(11): p. 2564-71.

43A. Roden, M., *Safety, tolerability and effects on cardiometabolic risk factors of empagliflozin monotherapy in drug-naive patients with type 2 diabetes: A double-blind extension of a Phase III randomized controlled trial.* Cardiovascular Diabetology 14 (1) (no pagination), 2015, 2015. **Article Number**: p. 154. Date of Publication: December 23.

44A. Rosenstock, J., et al., *Efficacy and safety of the dipeptidyl peptidase-4 inhibitor sitagliptin added to ongoing pioglitazone therapy in patients with type 2 diabetes: a 24-week, multicenter, randomized, double-blind, placebo-controlled, parallel-group study.* Clin Ther, 2006. **28**(10): p. 1556-68.

45A. Rosenstock, J., et al. (2009) *Effect of saxagliptin monotherapy in treatment-naive patients with type 2 diabetes*. Current Medical Research & Opinion **25**, 2401-2411.

46A. Rosenstock, J., C. Wilson, and P. Fleck, *Alogliptin versus glipizide monotherapy in elderly type 2 diabetes mellitus patients with mild hyperglycaemia: A prospective, double-blind, randomized, 1-year study.* Diabetes, Obesity and Metabolism, 2013. **15**(10): p. 906-914.

47A. Ross, S.A., et al., *Efficacy and safety of linagliptin 2.5mg twice daily versus 5mg once daily in patients with type 2 diabetes inadequately controlled on metformin: A randomised, double-blind, placebo-controlled trial.* Current Medical Research and Opinion, 2012. **28**(9): p. 1465-1474.

48A. Raz, I., et al., *Incidence of pancreatitis and pancreatic cancer in a randomized controlled multicenter trial (SAVOR-TIMI 53) of the dipeptidyl peptidase-4 inhibitor saxagliptin.* Diabetes care, 2014. **37**: p. 2435-41.

49A. Vilsboll, T., et al., *Efficacy and safety of sitagliptin when added to insulin therapy in patients with type 2 diabetes.* Diabetes, Obesity and Metabolism, 2010. **12**(2): p. 167-177.

50A. Wainstein, J., et al., *Initial therapy with the fixed-dose combination of sitagliptin and metformin results in greater improvement in glycaemic control compared with pioglitazone monotherapy in patients with type 2 diabetes.* Diabetes, Obesity and Metabolism, 2012. **14**(5): p. 409-418.

51A. White, W.B., et al., *Alogliptin after acute coronary syndrome in patients with type 2 diabetes.* N Engl J Med, 2013. **369**(14): p. 1327-35.

52A. Goldstein, B.J., et al., *Effect of initial combination therapy with sitagliptin, a dipeptidyl peptidase-4 inhibitor, and metformin on glycemic control in patients with type 2 diabetes.* Diabetes Care, 2007. **30**(8): p. 1979-87.

53A. Pratley, R.E., P. Fleck, and C. Wilson, *Efficacy and safety of initial combination therapy with alogliptin plus metformin versus either as monotherapy in drug-naive patients with type 2 diabetes: A randomized, double-blind, 6-month study.* Diabetes, obesity & metabolism, 2014. **16**: p. 613-21.

54A. Takihata, M., et al., *Comparative study of sitagliptin with pioglitazone in Japanese type 2 diabetic patients: The COMPASS randomized controlled trial.* Diabetes, Obesity and Metabolism, 2013. **15**(5): p. 455-462.

55A. Gallwitz, B., et al., *2-year efficacy and safety of linagliptin compared with glimepiride in patients with type 2 diabetes inadequately controlled on metformin: a randomised, double-blind, non-inferiority trial.* Lancet, 2012. **380**(9840): p. 475-83.

56A. Rosenstock, J., et al., *Dual add-on therapy in type 2 diabetes poorly controlled with metformin monotherapy: a randomized double-blind trial of saxagliptin plus dapagliflozin addition versus single addition of saxagliptin or dapagliflozin to metformin.* Diabetes Care, 2015. **38**(3): p. 376-83.

57A. Hirose, *Efficacy and Safety of Vildagliptin as an Add-on to Insulin with or without Metformin in Japanese Patients with Type 2 Diabetes Mellitus: A 12-week, Double-Blind, Randomized Study.* Diabetes Therapy, 2015. **6**(4): p. 559-571.

58A. Mita, T., *Sitagliptin attenuates the progression of carotid intima-media thickening in insulin-treated patients with type 2 diabetes: The sitagliptin preventive study of intima-media thickness evaluation (SPIKE): A randomized controlled trial.* Diabetes Care, 2016. **39**(3): p. 455-464.

59A. Hollander, P.L., et al. *Safety and efficacy of saxagliptin added to thiazolidinedione over 76 weeks in patients with type 2 diabetes mellitus*. Diab Vasc Dis Res,2011.**8**(2): p.125-135.

60A. Charbonnel, B., et al. *Efficacy and safety over 26 weeks of an oral treatment strategy including sitagliptin compared with an injectable treatment strategy with liraglutide in patients with type 2 diabetes mellitus inadequately controlled on metformin: a randomised clinical trial*. Diabetologia, 2013. **56**(7):p. 1503-1511.

61A. Seck, T., et al. *Safety and efficacy of treatment with sitagliptin or glipizide in patients with type 2 diabetes inadequately controlled on metformin: a 2-year study*. International Journal of Clinical Practice, 2010. **64**(5):p. 562-576.

62A. Weinstock, R.S., et al. *Safety and efficacy of once-weekly dulaglutide versus sitagliptin after 2 years in metformin-treated patients with type 2 diabetes (AWARD-5): a randomized, phase III study*. Diabetes Obes Metab, 2015. **17**(9):p. 849-858.

63A. Roden, M., et al. *Empagliflozin monotherapy with sitagliptin as an active comparator in patients with type 2 diabetes: a randomised, double-blind, placebo-controlled, phase 3 trial*. The Lancet Diabetes & Endocrinology, 2013. **1**(3):p. 208-219.

64A. Takeshita, Y., et al. *Vildagliptin vs liraglutide as a second-line therapy switched from sitagliptin-based regimens in patients with type 2 diabetes: A randomized, parallel-group study*. J Diabetes Investig, 2015. **6**(2):p. 192-200.

65A. Ma, Y., et al. *Clinical pharmacology of albiglutide, a GLP-1 receptor agonist*. Postgrad Med, 2014. **126**(7):p. 84-97.

66A. Moses, R. G., et al. *A randomized clinical trial evaluating the safety and efficacy of sitagliptin added to the combination of sulfonylurea and metformin in patients with type 2 diabetes mellitus and inadequate glycemic control*. J Diabetes, 2016. **8**(5):p. 701-711.

67A. *Metformin Add-on Regimen Comparison Study in Japanese Participants With Type 2 Diabetes Mellitus (MK-0431A-136)*.<http://ClinicalTrials.gov/show/NCT01545388>.

68A. *Efficacy and Safety of Albiglutide in Treatment of Type 2 Diabetes.* <http://ClinicalTrials.gov/show/NCT00838903>.

69A. Bosi, E., et al. *Alogliptin as a third oral antidiabetic drug in patients with type 2 diabetes and inadequate glycaemic control on metformin and pioglitazone: a 52-week, randomized, double-blind, active-controlled, parallelgroup study.* Diabetes Obes Metab, 2011. **13**(12):p.1088–1096.

70A. Bosi, E., et al. *Vildagliptin plus metformin combination therapy provides superior glycaemic*

*control to individual monotherapy in treatment-naive patients with type 2 diabetes mellitus.* Diabetes Obes Metab, 2009. **11**(5):p.506–515.

71A. Fonseca, V., et al. *Addition of vildagliptin to insulin improves glycaemic control in type 2 diabetes.* Diabetologia, 2007. **50**(6):p.1148–1155.

72A. Pan, C., et al. *Efficacy and tolerability of vildagliptin as add-on therapy to metformin in Chinese patients with type 2 diabetes mellitus.* Diabetes Obes Metab, 2012. **14**(8):p.737–744.

73A. Scherbaum, W.A., et al. *Efficacy and tolerability of vildagliptin in drug-naive patients with type 2 diabetes and mild hyperglycaemia* .* Diabetes Obes Metab, 2008. **10**(8):p.675–682.

74A. McGill, J.B., et al.,*Linagliptin added to sulphonylurea in uncontrolled type 2 diabetes patients with moderate-to-severe renal impairment.* Diab Vasc Dis Res, 2013. **11**(1):p.34-40.

75A. Ferrannini, E., et al. *Long-term safety and efficacy of empagliflozin, sitagliptin, and metformin: an active-controlled, parallel-group, randomized, 78-week open-label extension study in patients with type 2 diabetes.* Diabetes Care, 2013. **36**(12):p.4015-21.
