# Supplementary material for: The effects of dipeptidyl peptidase-4 inhibitors on bone fracture among patients with type 2 diabetes mellitus: A network meta-analysis of randomized controlled trials
Source: PLoS One. 2017 Dec 5;12(12):e0187537. doi: 10.1371/journal.pone.0187537 (PMC5716604; doi:10.1371/journal.pone.0187537)
Supplement: S2 Table — Note: GLP-1RAs: (Glucagon-like peptide-1) receptor agonists; SGLT-2: Sodium-Glucose co-Transporter 2; Met: metformin; SU: sulphanylureas; TZD: thiazolidinediones. (DOCX) [file pone.0187537.s003.docx]

**S2 Table. Node split method for inconsistence check in network**

| Comparison | Direct | | Indirect | | Difference | | P |
| --- | --- | --- | --- | --- | --- | --- | --- |
|  | Coef. | S.E. | Coef. | S.E. | Coef. | S.E. |  |
| Alogliptin vs Placebo | 0.6534673 | 0.3109991 | 0.8144742 | 0.6601806 | -0.1610069 | 0.7297735 | 0.825 |
| Alogliptin vs SU | 0.0835191 | 0.5723433 | 0.1079584 | 0.4610698 | -0.0244394 | 0.7349572 | 0.973 |
| Alogliptin vs TZD | 2.047849 | 1.587327 | -0.522498 | 0.9853233 | 2.570347 | 1.921661 | 0.181 |
| GLP-1RAs vs Placebo | 1.09595 | 2.003285 | 0.3375515 | 0.7256537 | 0.7583984 | 2.130925 | 0.722 |
| GLP-1RAs vs Sitagliptin | 0.0217796 | 0.6840839 | -0.7895481 | 1.297498 | 0.8113277 | 1.353374 | 0.549 |
| GLP-1RAs vs SU | 0.986378 | 1.348668 | -0.4452502 | 0.7537319 | 1.431628 | 1.444081 | 0.322 |
| GLP-1RAs vs Vildagliptin | -1.147798 | 1.643001 | 0.8593131 | 0.9964404 | -2.007111 | 1.921548 | 0.296 |
| Linagliptin vs Placebo | -0.0617424 | 0.3599897 | -0.3362207 | 0.6502259 | 0.2744783 | 0.743227 | 0.712 |
| Linagliptin vs Met | -0.6665337 | 1.515245 | 0.0085266 | 0.9865694 | -0.6750603 | 1.808114 | 0.709 |
| Linagliptin vs SU | -0.8161314 | 0.603085 | -0.6419117 | 0.4860005 | -0.1742197 | 0.7745373 | 0.822 |
| Met vs Placebo | -0.3721903 | 1.63981 | 0.2058363 | 0.924976 | -0.5780266 | 1.8827 | 0.759 |
| Met vs Sitagliptin | -0.5276206 | 1.111897 | -0.415954 | 1.157406 | -0.1118252 | 1.604967 | 0.944 |
| SGLT2 vs Placebo | 0.7889057 | 0.7183029 | 0.1302893 | 0.8730541 | 0.6586164 | 1.144333 | 0.565 |
| SGLT2 vs Saxagliptin | 0.9528541 | 1.554595 | 0.573083 | 0.5990864 | 0.3797711 | 1.666035 | 0.820 |
| SGLT2 vs Sitagliptin | -0.2952572 | 0.6910753 | 0.6399432 | 1.126847 | -0.9352004 | 1.392352 | 0.502 |
| Saxagliptin vs Placebo | -0.0972671 | 0.139485 | -0.1755116 | 0.6549875 | 0.0782445 | 0.6696751 | 0.907 |
| Saxagliptin vs SU | -0.6992461 | 0.6144716 | -0.6797541 | 0.3743923 | -0.0194921 | 0.7195446 | 0.978 |
| Sitagliptin vs Placebo | 0.5964493 | 0.3482673 | 0.4291936 | 0.4901045 | 0.1672557 | 0.6019881 | 0.781 |
| Sitagliptin vs SU | -0.0785488 | 0.4025058 | 0.0084987 | 0.4974427 | -0.0870476 | 0.639891 | 0.892 |
| Sitagliptin vs TZD | -0.5394459 | 0.9449181 | 1.308341 | 1.328626 | -1.847787 | 1.630366 | 0.257 |
| SU vs Placebo | -0.3470254 | 1.334682 | 0.6419721 | 0.3152285 | -0.9889975 | 1.379773 | 0.474 |
| TZD vs Placebo | -0.4542078 | 1.589651 | 0.8078304 | 0.9480594 | -1.262038 | 1.90799 | 0.508 |
| Vildagliptin vs Placebo | -0.1368283 | 0.6683724 | 1.870283 | 1.801566 | -2.00711 | 1.921552 | 0.296 |

Note: GLP-1RAs: (Glucagon-like peptide-1) receptor agonists; SGLT-2: Sodium-Glucose co-Transporter 2; Met: metformin; SU: sulphanylureas; TZD: thiazolidinediones.
