# Supplementary material for: The effects of dipeptidyl peptidase-4 inhibitors on bone fracture among patients with type 2 diabetes mellitus: A network meta-analysis of randomized controlled trials
Source: PLoS One. 2017 Dec 5;12(12):e0187537. doi: 10.1371/journal.pone.0187537 (PMC5716604; doi:10.1371/journal.pone.0187537)
Supplement: S3 Table — Note: NA: not available. GLP-1RAs (Glucagon-like peptide-1) receptor agonists; SGLT-2: Sodium-Glucose co-Transporter 2; Met: metformin; SU: sulphanylureas; TZDs: thiazolidinediones. (DOCX) [file pone.0187537.s004.docx]

**S3 Table. Odds ratio (OR) with 95% confidence interval (CI) of NMA for bone fracture events based on a frequentist framework when compared with placebo.**

| **Characteristics** | **Alogliptin** | **Sitagliptin** | **Linagliptin** | **Saxagliptin** | **Vildagliptin** | **SU** | **Met** | **GLP-1RAs** | **SGLT-2** | **TZDs** |
| --- | --- | --- | --- | --- | --- | --- | --- | --- | --- | --- |
| **All trials** | **0.51**  **(0.29,0.88)** | 0.58 (0.33,1.02) | 1.13  (0.61,2.10) | 1.11 (0.85,1.44) | 0.90  (0.26,3.07) | 0.56 (0.31,1.01) | 0.94  (0.19,4.54) | 0.65  (0.17,2.49) | 0.59 (0.20,1.74) | 0.63 (0.13,2.96) |
| **Double blind trials** | **0.51 (0.29,0.90)** | 0.58 (0.33,1.03) | 1.11 (0.59,2.08) | 1.11 (0.85,1.45) | 1.15  (0.31,4.25) | 0.56 (0.31,1.02) | 1.11 (0.18,7.05) | 0.81 (0.16,4.03) | 0.79 (0.25,2.46) | 0.64 (0.12,3.36) |
| **Mean age** |  |  |  |  |  |  |  |  |  |  |
| **＜60years** | 0.52 (0.21,1.24) | **0.52 (0.28,0.97)** | 1.02 (0.47,2.21) | 1.27 (0.58,2.76) | 1.24  (0.25,6.17) | 0.54 (0.27,1.11) | 1.03 (0.16,6.56) | 0.30 (0.06,1.49) | 0.57 (0.19,1.70) | 0.79 (0.10,6.02) |
| **≥60years** | **0.49 (0.24,1.00)** | 0.74 (0.14,3.92) | 1.32 (0.44,4.01) | 1.08 (0.81,1.43) | 1.02 (0.13,7.89) | 0.46 (0.12,1.73) | 0.68 (0.03,16.16) | 3.44 (0.25,47.52) | NA | 0.75 (0.01,53.99) |
| **Trial duration** |  |  |  |  |  |  |  |  |  |  |
| **24-52weeks** | 0.30 (0.06,1.68) | 0.58 (0.24,1.41) | 1.01 (0.33,3.10) | 1.06 (0.38,3.01) | 1.24  (0.25,6.16) | 0.29 (0.02,3.73) | 1.11 (0.16,7.44) | 0.08 (0.00,1.81) | 0.77 (0.16,3.85) | 0.68 (0.10,4.47) |
| **≥52weeks** | **0.55 (0.30,0.98)** | 0.56 (0.26,1.24) | 1.32 (0.59,2.97) | 1.12 (0.85,1.47) | 0.32  (0.01,7.88) | 0.60 (0.31,1.16) | 0.68 (0.03,14.74) | 0.79 (0.15,4.25) | 0.46 (0.11,2.01) | 0.37 (0.02,6.69) |
| **T2DM duration** | |  |  |  |  |  |  |  |  |  |
| **1-5years** | 0.38 (0.03,4.58) | 0.49 (0.13,1.85) | NA | 1.32 (0.20,8.55) | 0.32  (0.01,7.88) | NA | 0.16 (0.01,5.27) | NA | NA | 0.17 (0.01,5.31) |
| **≥5years** | **0.52 (0.29,0.94)** | 0.52 (0.22,1.25) | NA | 1.08 (0.82,1.42) | 1.48  (0.35,6.22) | 0.54 (0.23,1.31) | NA | 0.42 (0.06,3.01) | 0.74 (0.15,3.68) | 2.06 (0.16,26.35) |
| **Background therapy** | |  |  |  |  |  |  |  |  |  |
| **With Met** | 0.50  (0.20,1.28) | **0.49 (0.24,0.99)** | 1.09  (0.46,2.57) | 1.10 (0.83,1.44) | 1.89 (0.30,12.06) | 0.56 (0.28,1.12) | NA | 0.46  (0.07,2.97) | 0.45 (0.11,1.84) | 0.56 (0.12,2.74) |
| **Without Met** | **0.48**  **(0.23,1.00)** | 1.12 (0.32,3.85) | 1.19  (0.48,2.96) | 1.31 (0.22,7.84) | 0.74  (0.10,5.36) | NA | 0.91  (0.10,8.52) | 1.20  (0.15,9.78) | NA | NA |
| **Monotherapy** | 0.36  (0.05,2.80) | 0.34 (0.06,1.97) | NA | 1.70 (0.08,35.92) | 0.32  (0.01,7.88) | 0.17  (0.02,1.51) | 0.58  (0.04,9.52) | NA | 0.64  (0.11,3.66) | NA |

Note: NA: not available. GLP-1RAs (Glucagon-like peptide-1) receptor agonists; SGLT-2: Sodium-Glucose co-Transporter 2; Met: metformin; SU: sulphanylureas; TZDs: thiazolidinedione.
